# Organic osmolytes preserve the function of the developing tight junction in ultraviolet B-irradiated rat epidermal keratinocytes

**DOI:** 10.1038/s41598-018-22533-0

**Published:** 2018-03-26

**Authors:** Cécile El-Chami, Iain S. Haslam, Martin C. Steward, Catherine A. O’Neill

**Affiliations:** 1 School of Biological Sciences, Division of Musculoskeletal & Dermatological Sciences, Faculty of Biology, Medicine and Health, University of Manchester, Oxford Road, Manchester, M13 9PT United Kingdom; 2 School of Medical Sciences, Division of Diabetes, Endocrinology and Gastroenterology, Faculty of Biology, Medicine and Health, University of Manchester, Oxford Road, Manchester, M13 9PT United Kingdom; 3 Present Address: Department of Biological Sciences, School of Applied Sciences, University of Huddersfield, Queensgate, Huddersfield, HD1 3DH United Kingdom

## Abstract

Epidermal barrier function is provided by the highly keratinised stratum corneum and also by tight junctions (TJs) in the granular layer of skin. The development of the TJ barrier significantly deteriorates in response to ultraviolet B radiation (UVB). Following exposure to UVB, keratinocytes accumulate organic osmolytes, which are known to preserve cell volume during water stress. Since TJs are intimately associated with control of water homeostasis in skin, we hypothesised that there may be a direct influence of osmolytes on TJ development. Exposure of rat epidermal keratinocytes (REKs) to a single dose of UVB reduced the function of developing TJs. This was concomitant with dislocalisation of claudin-1 and claudin-4 from the keratinocyte plasma membrane, phosphorylation of occludin and elevation of reactive oxygen species (ROS). In the presence of organic osmolytes, these effects were negated but were independent of the effects of these molecules on cell volume, elevation of ROS or the gene expression of TJ proteins. These data suggest that organic osmolytes affect TJs via post-translational mechanism(s) possibly involving protection of the native conformation of TJ proteins.

## Introduction

During the process of evolution, a major requirement for terrestrial organisms was the development of mechanisms to reduce water loss. Of importance in this regard was the evolution of the skin, the epidermis of which provides a tough, waterproof barrier to limit water loss^1,2^. A number of water controlling structures are present in the epidermis. In particular, the stratum corneum, which is the outermost layer of the epidermis and is the result of keratinocyte terminal differentiation, is probably the best characterised^3–5^. The stratum corneum is composed of 18–20 layers of hexagonal-shaped, flattened, anucleated cells called corneocytes, which are embedded in a lipid matrix in what is commonly referred to as a ‘bricks-and-mortar’ structure^3–5^.The appropriate deposition of lipids in the extracellular space and their proper organization in bilayer membranes to form the intercellular lipid lamellar structures in the stratum corneum is very important for the formation of an effective barrier to transepidermal water loss (TEWL)^6,7^. In the human stratum corneum, lipids are tightly packed in an orthorhombic lateral arrangement which permit low levels of water diffusion through the intercellular space between corneocytes, and have an effective role in providing a barrier against water loss^6,7^. Furthermore, the lipid polar heads in the lamellar structures retain water molecules by forming hydrogen bonds^3,8^.

More recently, tight junctions (TJs) have been shown to play an important role in sealing the gaps between cells to prevent water loss, therefore controlling the extracellular passage of molecules, ions and water^9–12^. TJs are multi-protein complexes located in the granular layer of the epidermis in human skin^13,14^. The main components of TJs are transmembrane proteins such as the claudin family, of which there are 27 isoforms in man, occludin, and cytoplasmic proteins such as ZO-1^15–17^. Claudins have a critical function in TJs and are the main determinants of the paracellular barrier characteristics in simple epithelia. Overexpression of claudin-1 results in an increase in transepithelial electrical resistance (TEER) with a reduction in the paracellular flux of FITC-dextran tracer in the Madin-Darby canine kidney (MDCK) cell line^18^. Moreover, overexpression of claudin-4 in TJs of submandibular gland cells^19^, and MDCK cells^20,21^ leads to an increase in TEER and a decrease in permeability to cations. Many studies have indicated that occludin is a primary TJ component, with overexpression of full length occludin in cultured MDCK cells increasing TEER^22,23^. A recent study showed that knock down of occludin in intestinal epithelial cells both *in vitro* and *in vivo* resulted in an increase in the flux of a macromolecular, paracellular probe across the intestinal epithelial TJ barrier^24^. However, the flux of ions across the TJ was not affected suggesting that occludin is a specific barrier to macromolecular transport^24^.

TJs have been shown to contribute significantly to a regulated and selective epidermal permeability barrier, and the claudin protein family is considered to be a key component of this structure^13,25,26^. For example, the claudin-1 knockout mouse dies 24 hours after birth due to excessive transepidermal water loss^25^. Studies performed in human epidermis and cultured human keratinocytes also reveal a direct contribution of claudin-1, claudin-4 and occludin to the passage of ions, intermediate-sized molecules and macromolecules^13,27–29^. Importantly, in keratinocytes, TJs also have a direct role in forming a barrier to water loss^29^.

Among the different environmental stressors that skin is exposed to on a daily basis, ultraviolet radiation (UVR) from sunlight is probably the most significant. Previous studies have shown that UVB exposure leads to reactive oxygen species (ROS) accumulation and increased oxidative stress in skin cells both *in vitro* and *in vivo*^30–33^. In normal skin, exposure to UVR causes epidermal permeability barrier disruption manifest by an increase in TEWL^14,34–37^. A number of studies have also shown that UVB irradiation increases penetration of biotinylated markers through TJs suggesting an impairment of their barrier function^14,36^. This is accompanied by mislocalisation of key TJ proteins^14,36^.

Following the disruption of the epidermal permeability barrier, extracellular water will leave the skin by diffusion to the external dry environment. As a result the extracellular fluid surrounding the epidermal keratinocytes will become hypertonic^38^. In response to hypertonicity, intracellular water will leave keratinocytes by osmosis^39^, leading to cell shrinkage. Maintaining a constant cell volume is crucial for normal cellular activities, such as growth, migration and the regulation of intracellular metabolism^40–42^. Cells, including keratinocytes, implement various strategies in order to regulate their cell volume and help retain intracellular water. One such strategy is the accumulation of organic osmolytes in response to cell shrinkage^40,41,43–46^.

Organic osmolytes can be grouped into three classes: (1) polyols (2) amino acid derivatives and (3) methylamines^40,43,47^. These are neutral (either zwitterionic or lacking charge at physiological pH), non-perturbing, “compatible” molecules. Unlike inorganic osmolytes such as sodium (Na^+^), potassium (K^+^), and chloride (Cl^-^) ions, which can interfere with electrical charge balance and the structure and function of proteins and nucleic acids^48^, organic osmolytes even at high concentrations (10–100 mM), do not significantly disturb membrane potential, enzyme activities or the ionic strength of the cytoplasm. Hence, organic osmolytes can counteract the damage that might be caused by osmotic stress without conferring any damage themselves^40,43,47,49^.

A small number of studies performed on cultured keratinocytes have revealed that normal human epidermal keratinocytes (NHEKs) increase the gene expression of the betaine/γ-amino-n-butyric acid (GABA) transporter (BGT-1), sodium/taurine transporter (TAUT) and sodium/*myo-*inositol cotransporter (SMIT) in response to both hypertonicity and doses of UVA and UVB radiation^44,45^. This results in an increased uptake of radiolabeled *myo-*inositol, taurine and betaine in these cells^44,45^. Another study performed in the immortal human keratinocyte line (HaCaT), has shown that these cells lose water and shrink after UVB exposure, which leads to an increase in BGT-1, TAUT and SMIT mRNA expression, resulting in an intracellular accumulation of their respective osmolytes^46^.

Although these studies suggest that keratinocytes possess an osmolyte strategy, the mechanisms regulating cellular water in skin are poorly characterised, and information is lacking as to how they function in concert with extracellular mechanisms to retain water levels and limit water loss. We hypothesize that organic osmolytes may interact with other epidermal structures such as the TJs responsible for the regulation of epidermal extracellular water loss. This study aimed to investigate the potential role of organic osmolytes in maintaining the integrity of developing TJs following UVB-induced disruption in the rat epidermal keratinocyte (REK) cell line.

## Results

### Organic osmolytes reduce cell shrinkage but have no effect on ROS levels in UVB-exposed REKs

**Figure 1:**
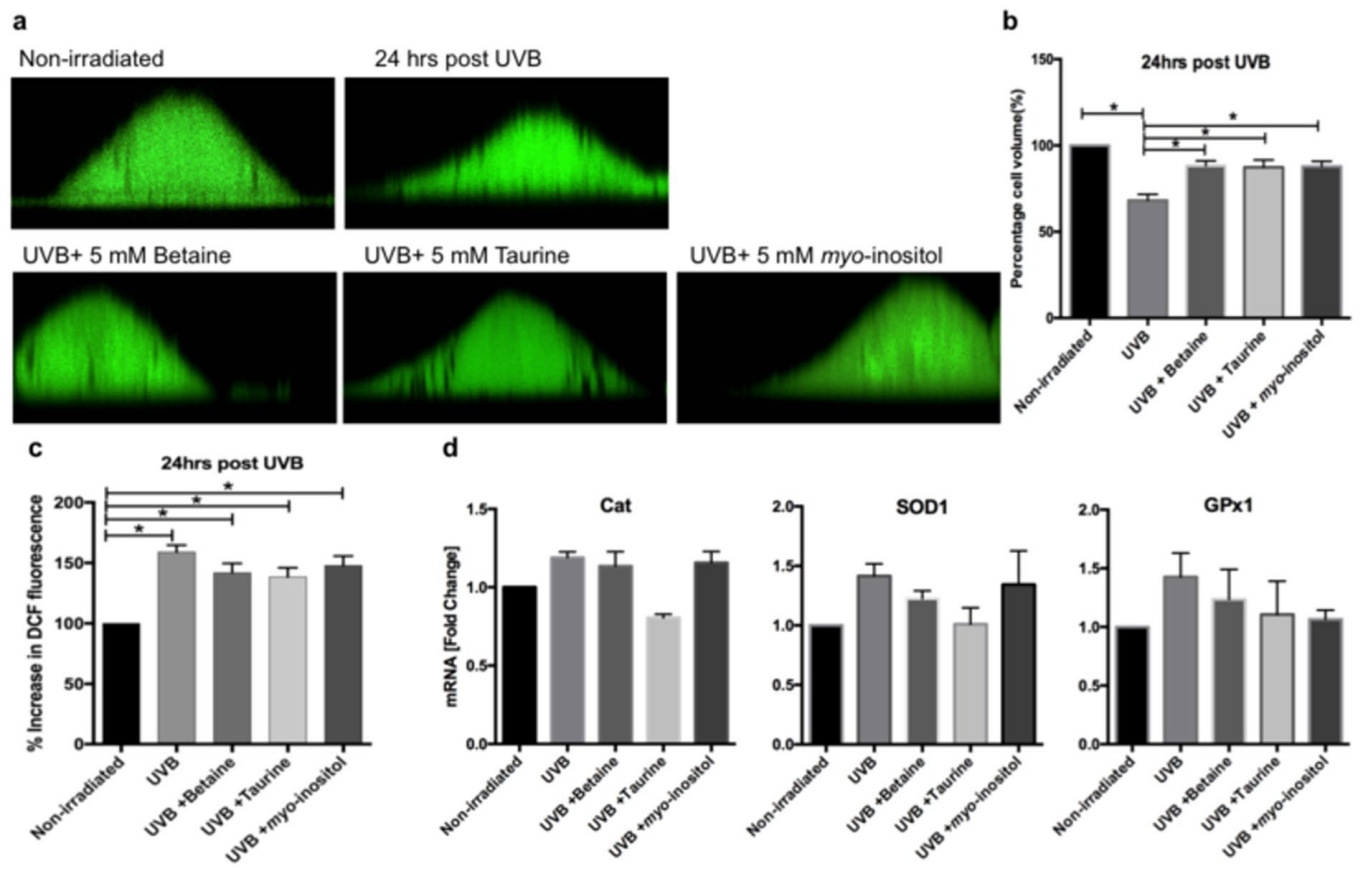
Effect of UVB on cell volume and ROS levels in REKs. (**a**) Images represent REKs under non-irradiated condition and 24 hours post UVB with and without organic osmolytes. (**b**) The cell volume of untreated and organic osmolyte- treated and UVB-exposed keratinocytes was calculated relative to non-irradiated keratinocyte volume and is presented as percentage cell volume (n = 4; mean ± SEM; *p < 0.05). (**c**) Increase in DCF fluorescence intensity corresponding to increase in ROS levels (n = 4; mean ± SEM; *p < 0.05). (**d**) Catalase (Cat), superoxide dismutase (SOD1) and glutathione peroxidase (GPx1) gene expression levels at 24 hours post UVB exposure in non-organic and organic osmolytes-treated keratinocytes (n = 3; mean ± SEM).

We first investigated the basic cellular responses of rat epidermal keratinocytes to exposure to a single, sublethal dose of 10 mJ/cm^2^ UVB (Supplementary Fig. S1). This resulted in a significant decrease in cell volume 24 hours post irradiation (Fig. 1a,b). This was accompanied by an elevation in the level of ROS as indicated by the significant increase in the fluorescence intensity of the 2′−7′- dichlorofluorescein (DCF) probe in irradiated keratinocytes compared with non-irradiated cells (Fig. 1c). However, there was a non-significant change in the gene expression of the antioxidant enzymes, superoxide dismutase 1 (SOD1), catalase (Cat) and glutathione peroxidase (GPx1).

We next tested the ability of organic osmolytes to mitigate keratinocyte cell shrinkage following UVB exposure by supplementing the culture medium with 5 mM betaine, taurine or *myo*-inositol directly after irradiation. However, first we confirmed that REK cells possess the necessary transporters for these osmolytes (supplementary Fig. S2).

UVB-exposed REKs treated with organic osmolytes shrank to a lesser extent than UVB-irradiated keratinocytes cultured without organic osmolytes (Fig. 1a and b). On the other hand, the presence of organic osmolytes did not reduce the raised ROS levels following irradiation (Fig. 1c), and although the gene expression of catalase, superoxide dismutase 1 showed a trend to decrease in the presence of taurine, it was not significant (Fig. 1d).

### Organic osmolytes mitigate the damaging effect of UVB on the permeability of developing TJs in REKs

**Figure 2:**
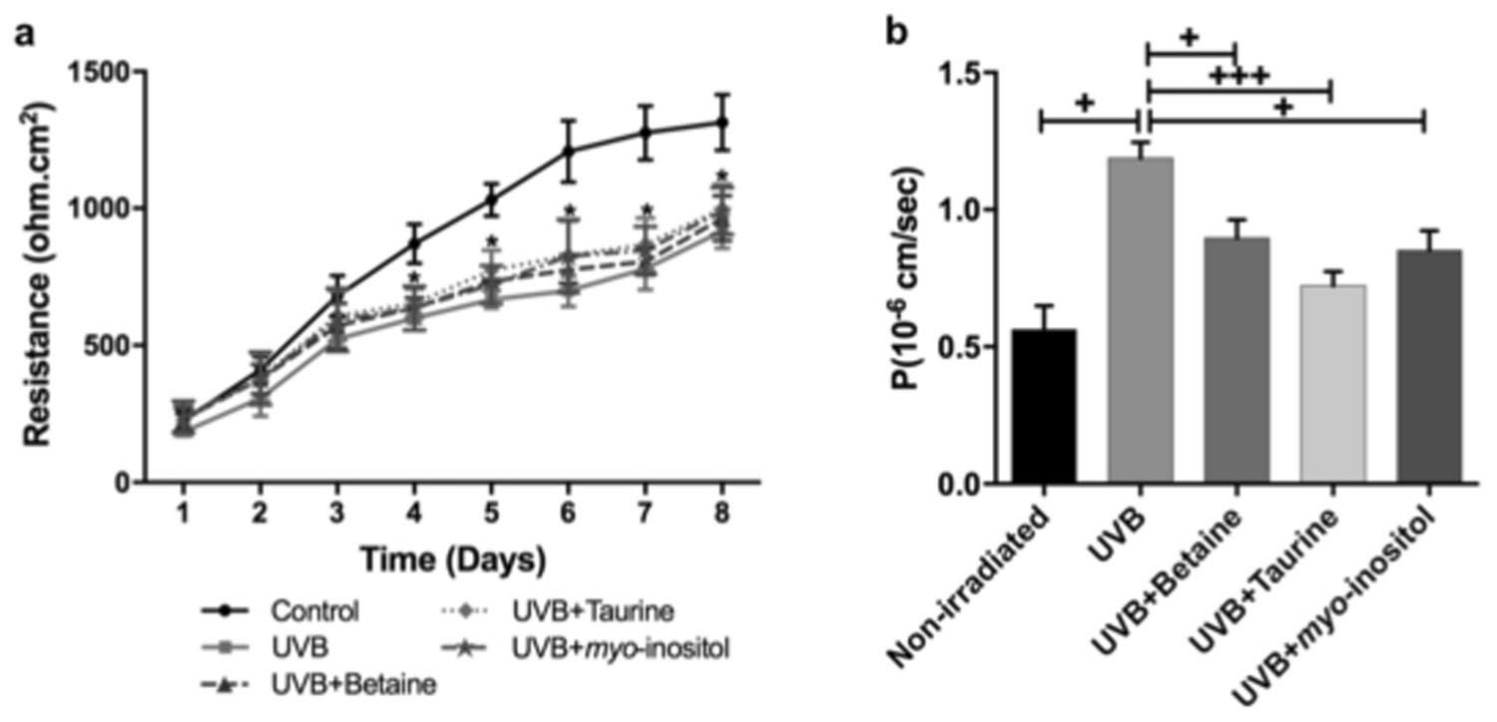
Effect of UVB and organic osmolytes on tight junction function in REKs. (**a**) TEER measurements following UVB exposure with and without organic osmolytes (n = 5; mean ± SEM; *P < 0.05 compared to control non-iradiated sample). (**b**) Permeability of 4 kDa FITC-dextran (FD4) 24 hours after UVB exposure (n = 5; mean ± SEM; ^+^p < 0.01 compared to non-irradiated cells).

The REK cell line is a spontaneously differentiating cell line, which can also stratify into skin equivalent type structures^50–52^. When plated on cell culture inserts, the TEER, a well established marker of TJ function^53^, increases with time (Fig. 2a). However, at time points beyond ~5 days post plating, the continued increase in TEER is largely due to cell stacking (data not shown). For this reason, we used earlier time points, when the cells are at monolayer to investigate the effects of UVB on the development of TJs.

Exposure of REKs to UVB resulted in a TEER measurement that was significantly lower than that in non-irradiated cells (Fig. 2a). Furthermore, the paracellular flux of a 4 kDa FITC-dextran tracer in UVB-exposed cell monolayers was significantly higher than in non-irradiated REK monolayers (Fig. 2b).

In UVB-exposed REKs cultured with 5 mM of betaine, taurine or myo-inositol, TEER measurements were reduced in a similar manner to those of UVB-exposed REK monolayers (Fig. 2a). In other words, the organic osmolytes had no protective effect on the UVB-evoked TEER changes. However, dextran permeability in UVB-irradiated REKs treated with organic osmolytes was significantly lower than that measured in UVB-irradiated cells without organic osmolytes (Fig. 2b). This decrease in dextran permeability appeared to be more prominent in taurine-treated cells.

Treatment of non-irradiated cells with 5 mM organic osmolytes had no effect on TEER (Supplementary Fig. S3a) or dextran permeability (Supplementary Fig. S3b).

### Organic osmolytes partially negate UVB-mediated dislocalisation of TJ proteins in REKs

**Figure 3:**
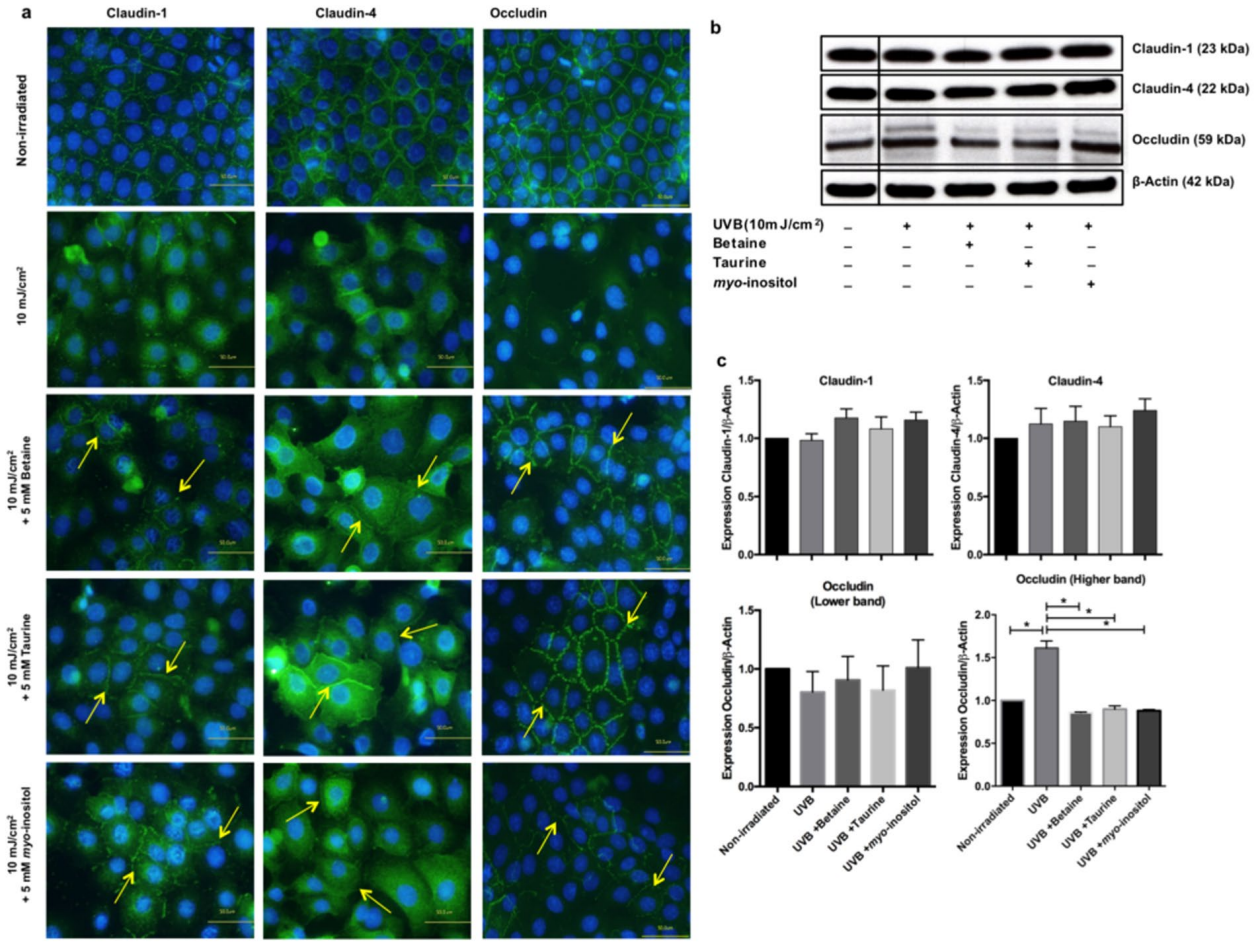
Effect of organic osmolytes on tight junction protein expression and localisation in UVB-irradiated REKs. (**a**) Immunostaining of claudin-1, claudin-4, and occludin at 24 hours after exposure to 10 mJ/cm^2^ of UVB in non-irradiated REKs and REKs supplemented with 5 mM organic osmolytes. Bar = 50μm. (**b**) Immunoblot of claudins-1, -4 and occludin in UVB-irradiated REK with and without organic osmolytes treatement. Full-length blots are presented in Supplementary Fig. S4. (**c**) Densitometric quantification showing the ratio of TJ protein expression to ß-actin protein expression (n = 3; mean ± SEM; *p < 0.05 compared to irradiated sample, non-supplemented with organic osmolytes).

Immunolocalisation of TJ proteins in non-irradiated cells showed a continuous network of claudins -1, -4 and occludin staining along the REKs plasma membrane (Fig. 3a). In contrast, in UVB-irradiated REKs, claudins-1, -4 and occludin were almost completely relocalised to the cell cytoplasm 24 hours post irradiation (Fig. 3a). There was no change in either the gene (Supplementary Fig. S4) or total protein expression of claudins-1, -4, or occludin at 24 hours after UVB irradiation (Fig. 3b,c, and Supplementary Fig. S5). However, there was a difference in the signal for occludin when investigated by immunoblotting. Occludin normally appears as a doublet, which reflects the multiple observations in the literature that this protein is phosphorylated^54–57^. In response to UVB, there was an increase in the phosphorylated form of occludin resulting in an increase in the density of the higher band on the immunoblot (Fig. 3b,c, and Supplementary Fig. S5)

In REKs exposed to UVB and treated with organic osmolytes, both claudins-1 and -4 were found both in the cytoplasm and also at the membrane although in a discontinuous pattern (Fig. 3a). The effects of the osmolytes appeared qualitatively to be the greatest for occludin. Treatment with the organic osmolytes maintained a continuous occludin staining pattern at the cell membrane. This was qualitatively much more noticeable with taurine (Fig. 3a). Organic osmolytes had no effect on the mRNA (Supplementary Fig. S4) and protein levels of TJ markers in irradiated REKs supplemented with organic osmolytes (Fig. 3b,c, and Supplementary Fig. S5). However, the density of the phosphorylated form of occludin, which increased upon irradiation, was lower in the presence of all of the organic osmolytes as confirmed by densitometric analysis of the immunoblot (Fig. 3b,c, and Supplementary Fig. S5).

### Cell shrinkage alone does not affect TJ proteins localisation in REKs

We next investigated the potential mechanisms underlying dysfunction of the developing TJs in response to UVB by investigating the effects of cell shrinkage and ROS (induced by H_2_O_2_), *per se* on keratinocytes.

**Figure 4:**
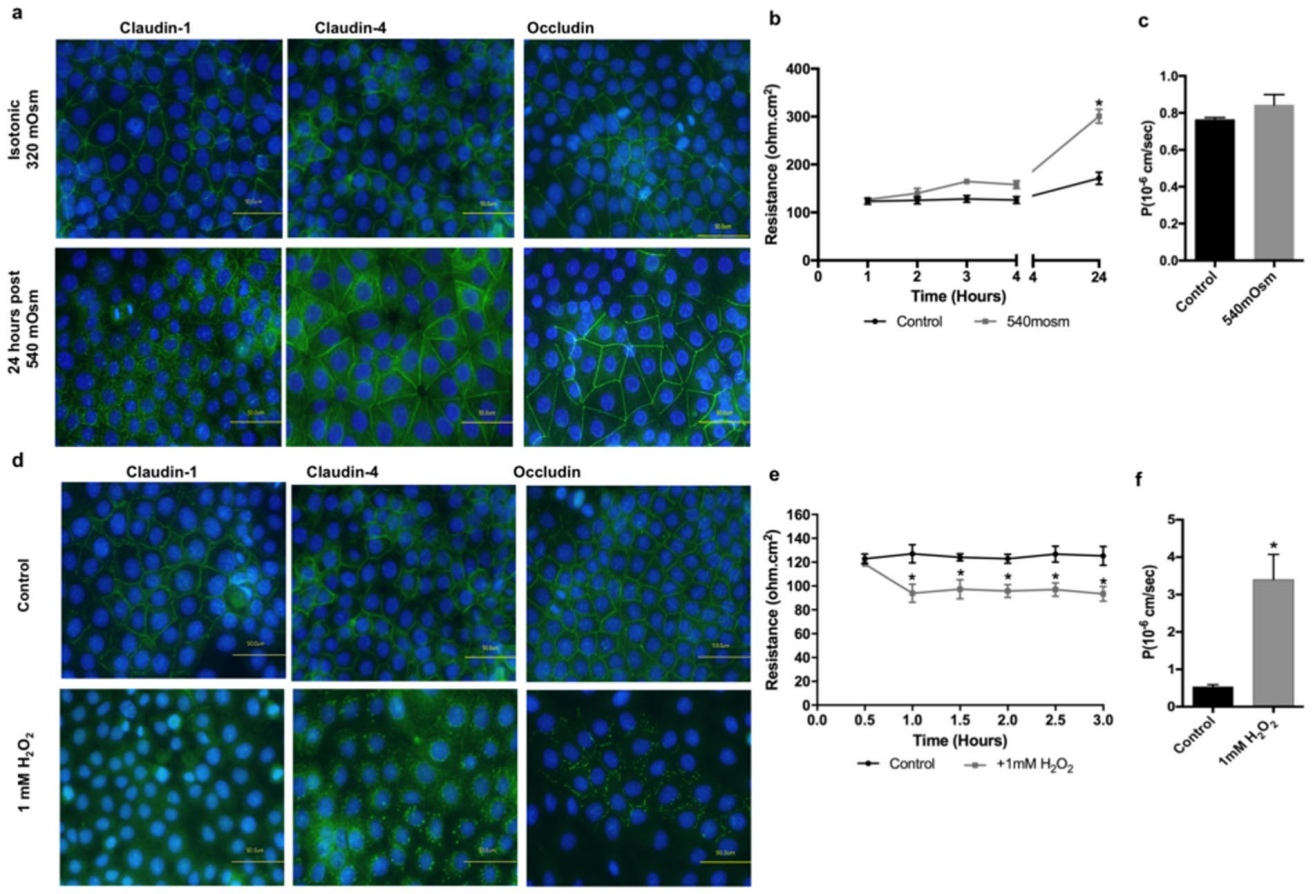
Effect of hyperosmotic stress and exogenous H_2_O_2_ on TJ structure and function in REKs. (**a**) Immunostaining of claudin-1, claudin-4, and occludin 24 hours post exposure to 540 mOsm culture medium. Bar = 50μm. (**b**) TEER measurements following hypertonic stress (n = 3; mean ± SEM; *P < 0.05 compared to control isotonic condition). (**c**) Permeability of 4 kDa FITC-dextran (FD4) 24 hours following hypertonic stress (n = 3; mean ± SEM; *p < 0.01 compared to control, isotonic condition). (**d**) Immunostaining of claudin-1, claudin-4, and occludin 24 hours post exposure to 1 mM H_2_O_2_. Bar = 50μm. (**e**) TEER measurements following application of H_2_O_2_ (n = 3; mean ± SEM; *P < 0.05 compared to control isotonic condition). (**f**) Permeability of 4 kDa FITC-dextran (FD4) 1 hour following application of H_2_O_2_ (n = 3; mean ± SEM; *p < 0.01 compared to control cells).

Hypertonic medium was used to induce keratinocyte shrinkage (Supplementary Fig. S6). However, the membranous staining pattern for claudin-1, claudin-4 and occludin was largely preserved, although small pools of claudins-1 and -4 were observed in the cytoplasm (Fig. 4a). Interestingly, claudin-4 gene and protein expression were upregulated in keratinocytes 24 hours following exposure to hypertonic medium (Supplementary Figs S6 and S7). TEER values recorded at the 24 hour time point were significantly higher in cells exposed to hypertonic stress compared to control cells (Fig. 4b). However dextran permeability was not affected by the exposure to hypertonic medium and was similar to the dextran permeability in control cells grown under normal isotonic conditions (Fig. 4c). Thus, hypertonicity *per se* did not reproduce the pattern of TJ dysfunction observed with UVB irradiation.

### Exogenous H_2_O_2_ causes TJ proteins delocalisation and reduction in the permeability of developing TJs

**Figure 5:**
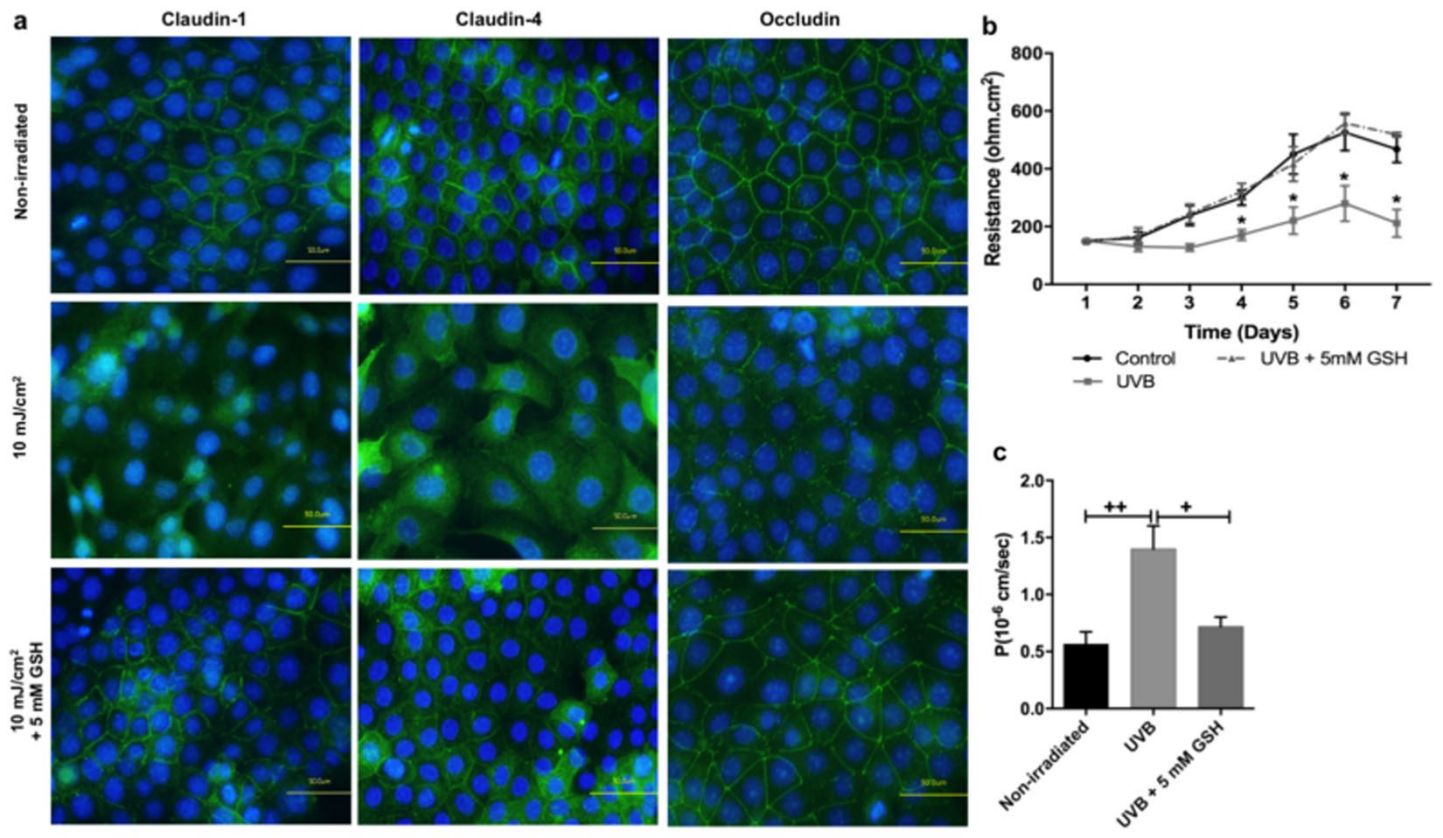
Effect of UVB on TJ structure and function in in the presence of GSH in REKs. (**a**) Immunostaining of claudin-1, claudin-4, and occludin at 24 hours after exposure to 10 mJ/cm^2^ of UVB in irradiated REKs and irradiated REKs supplemented with 5 mM GSH. Bar = 50μm. (**b**) TEER measurements following UVB exposure (n = 3; mean ± SEM; *P < 0.05 compared to control, non-iradiated sample). (**c)** Permeability of 4 kDa FITC-dextran (FD4) 24 hours following UVB exposure (n = 3; mean ± SEM; *p < 0.01 compared to irradiated cells).

Immunostaining revealed that exposure to hydrogen peroxide (H_2_O_2_) caused dislocalisation of claudin-1 to the cytoplasm. Similarly, claudin-4 was found to be cytoplasmic after exposure to H_2_O_2_, with the formation of what appeared to be autophagosome-like stuctures around the nuclei. Occludin showed fragmented membranous staining after H_2_O_2_ exposure (Fig. 4d). TEER measurements were significantly lower in cells exposed to 1 mM H_2_O_2_ compared to control cells (Fig. 4e). In line with the TEER measurement, the paracellular flux of the 4-kDa FITC-dextran tracer was higher in cells exposed to H_2_O_2_ compared to control cells (Fig. 4f), which indicates a disruption in the permeability of developing TJ following exposure to exogenous H_2_O_2_. Interestingly, in cells pre-treated with 5 mM glutathione (GSH), the UVB-induced decrease in TEER and increase in dextran permeability were significantly inhibited (Fig. 5a,b). Assessment of TJ proteins localisation in UVB-irradiated REKs monolayers pretreated with GSH revealed membraneous staining of claudin-1, claudin-4 and occludin, suggesting that the UVB-evoked elevation in ROS is largely responsible for TJ protein dysfunction (Fig. 5c).

**Figure 6:**
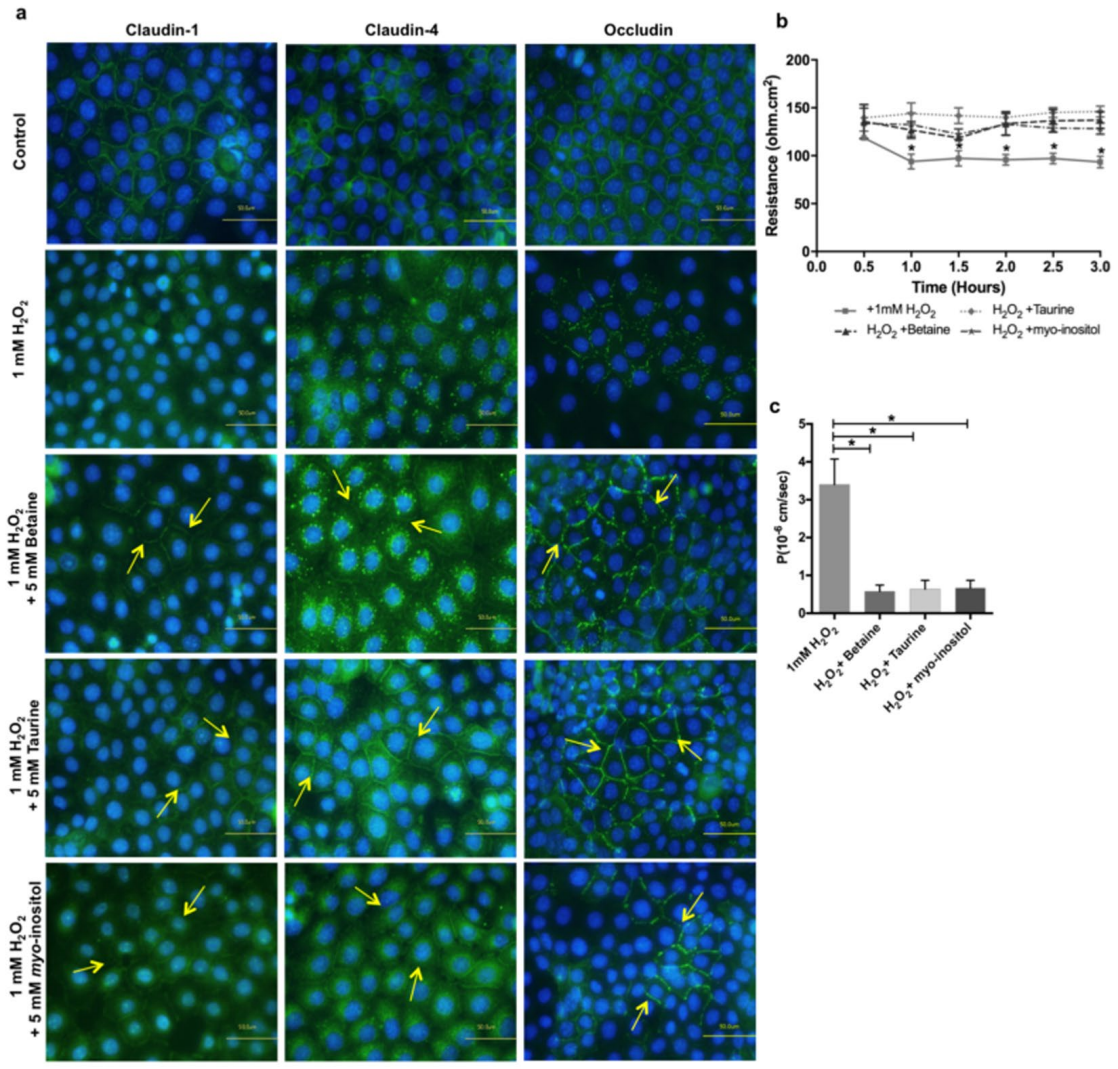
Effect of organic osmolytes on tight junction function and structure in H_2_O_2_-treated REKs. (**a**) Immunostaining of claudin-1, claudin-4, and occludin in organic osmolytes supplemented REKs at 1 hour post exposure to H_2_O_2_. Bar = 50μm. (**b**) TEER measurements of cells exposed to H_2_O_2_ and supplemented with organic osmolytes (n = 3; mean ± SEM *p < 0.05 compared to H_2_O_2_-exposed sample, non-supplemented with organic osmolytes). (**c**) Permeability of 4 kDa FITC-dextran (FD4) 1 hour following H_2_O_2_ exposure (n = 3; mean ± SEM; *p < 0.01 compared to H_2_O_2_-exposed sample, non-supplemented with organic osmolytes).

When H_2_O_2_-exposed cells were supplemented with betaine, taurine or myo-inositol, claudin-1, claudin-4 and occludin were preserved at the cell membrane. However, claudin-4 was still detectable in autophagosomes-like structures in betaine-supplemented cells (Fig. 6a).

The application of H_2_O_2_ caused a decrease in claudin-1, claudin-4 and occludin gene expression levels and this change was not mitigated by the presence of the organic osmolytes (Supplementary Figs S8 and S9). Only claudin-1 protein expression was reduced following H_2_O_2_ exposure and the presence of organic osmolytes could not negate this change (Supplementary Figs S8 and S9). Although total occludin protein expression was not modified following exposure to H_2_O_2_, occludin was found to have redistributed between high and low molecular weight forms (Supplementary Figs S8 and S9). Interestingly, in cells supplemented with organic osmolytes, total occludin protein expression was significantly higher than in non-osmolyte treated cells. Moreover treatment with betaine and taurine showed an increase in the higher molecular weight occludin band intensity but this upregulation was not significant compared to non-osmolyte treated cells (Supplementary Figs S8 and S9).

The presence of betaine, taurine and *myo*-inositol prevented the H_2_O_2_-induced reduction in TJ permeability barrier function (Fig. 6b,c). This was not accompanied by any reduction in ROS levels, or changes to the levels of antioxidant enzymes (Supplementary Fig. S10).

## Discussion

The present study is the first to investigate the effects of the organic osmolytes betaine, taurine and *myo-*inositol on the expression and function of developing TJs in keratinocytes. However, before discussing the roles of the osmolytes, it is first necessary to draw some conclusions as to the effects of UVB irradiation on TJs, which is itself, an under-researched area of investigation.

Many reports have described the damaging effect of UVR on the epidermal permeability barrier^31,34,58^. These studies have largely looked at the effects of UVR on the stratum corneum and very few studies have reported its damaging effect on TJ structure and function^14^. In the present study, and in line with previous results obtained with cultured normal human epidermal keratinocytes^14^, the permeability barrier of developing TJs in REK monolayers was reduced at 24 hours after UVB irradiation. The underlying mechanism likely involves relocalisation of TJ proteins from the TJ complexes, because the staining patterns of claudin-1, claudin-4 and occludin were dislocalised 24 hours after irradiation whereas no change was noted in either mRNA or total protein levels. Thus, the effect of UVB on TJs is arguably post-translational, at least at the time point used in this experiment.

Currently, it remains unclear how UVB affects TJ development, however a number of possibilities exist: UVB either damages TJ proteins directly, or changes the physiology of keratinocytes in a manner that induces TJ protein dislocalisation.

A previous study performed on HaCaT cells showed that these cells shrink in response to UVB exposure^46^, therefore it is possible that it is the change in keratinocyte volume that leads to disruption of TJ organization. TJ structures are linked to the actin-cytoskeleton via a number of TJ proteins such as occludin and the ZO proteins^59^, and changes in cell volume are found to have a major impact on the microfilament network organization of the cytoskeleton^60^. Moreover a previous study has shown that actin depolymerization induces endocytosis of TJ components^61^. Although our cell volume measurements showed that UVB caused keratinocytes to shrink, in a separate experiment shrinkage *per se* did not cause TJ protein dislocation. In line with previous finding in kidney cells, hyperosmotic conditions altered TJ proteins expression at the transcriptional and translational levels, specifically the induction of claudin-4 expression. In addition, hyperosmotic conditions reduced the permeability of TJs to ions, which is also in agreement with other studies^62–64^. Therefore it is possible that cell shrinkage induced by different stressors (in this case UVB and mannitol-induced hypertonicity) might cause different cellular responses. In our experiments, the cell shrinkage caused by UVB does not appear to be responsible for TJ proteins dislocalisation, hence UVB is affecting these structures via a different mechanism.

Besides causing alterations in cell volume, previous studies have shown that UVB exposure leads to ROS accumulation and increases oxidative stress in skin cells both *in vitro* and *in vivo*^30–33^. ROS production is known to modify proteins by inducing thiol oxidation, phosphorylation, carbonylation and nitration^65,66^. These post-translational modifications may be involved in the ROS-induced disruption of TJs in REK monolayers^67^. The degree of phosphorylation of TJ proteins is crucial for the maintenance of their function^54,68,69^. In normal epithelia, very low levels of phosphorylated occludin are detected^57^. However, during the disruption of TJs by, for example, oxidative stress, occludin phosphorylation is found to increase, which attenuates its interaction with ZO-1 and ZO-2/3, leading to the dissociation of the occludin-ZO complex from the intercellular junctions, and disruption of the paracellular barrier^57,70^. Our experiments showed increased density of the higher molecular weight band of occludin in response to UVB, suggesting that increased occludin phosphorylation might be part of the mechanism by which the developing TJ barrier is disrupted following irradiation. Generation of ROS in response to UVB exposure may be what is driving changes to the phosphorylation state of occludin, given that treatment of cells with H_2_O_2_ produced similar effects. Pretreatment with 5 mM GSH was shown to mitigate against the UVB-induced damage to TJ structure and function, which further supports the role of ROS in TJ disruption following acute UVB exposure.

The current study is the only one to our knowledge to demonstrate the ability of organic osmolytes to maintain keratinocyte volume following UVB irradiation. Furthermore organic osmolytes also maintain claudin-1, claudin-4 and occludin localisation at the TJ sites without any effect on their gene or protein expression in UVB-irradiated keratinocytes. More importantly, the reduction in the level of phosphorylated occludin following treatment with organic osmolytes, may explain the preservation of occludin at the cell-cell borders. The conservation of TJ protein membranous staining in organic osmolyte-treated cells was complemented by a significant reduction in dextran permeability without any effect on TEER. This suggests that the two paracellular pathways through the TJs, i.e. the charge-selective pore pathway that allows passage of small ions and uncharged molecules (reflected by TEER), and the leak pathway that allows flux of larger macromolecules^71,72^, are differentially regulated by osmolytes in REK cells. Previous work suggests that the structural basis of the leak pathway is associated with occludin^24,73^. Thus, the observation that occludin was post translationally modified by UVB, and that this modification was negated by osmolytes, is in keeping with the preservation of the leak pathway by osmolytes via occludin regulation. This finding not only further supports the importance of occludin in the regulation of the leak pathway flux but more importantly, it highlights the importance of organic osmolytes in maintaining TJ integrity thereby regulating the leak pathway.

Treating irradiated cells with taurine resulted in a noticeable preservation of membranous staining of occludin and also appeared to be more effective in reducing the elevated dextran permeability compared to betaine or myo-inositol. This osmolyte may therefore be of particular importance for the UVB response of keratinocytes. Although no previous studies have localised taurine in human skin, it was found to be highly concentrated in the granular and spinous layer of normal rat and dog epidermis^74^. The existence of taurine in normal skin further supports the argument that this osmolyte may be important in the physiological response to UVB.

Taken together, our data suggest that UVB-induced TJ disruption is to a large extent due to elevation of ROS levels. Furthermore, since the presence of betaine, taurine or myo-insitol following UVB and H_2_O_2_ exposure did not reduce the level of ROS, and had no effect on the mRNA level of the antioxidant enzymes, we suggest that organic osmolytes protect TJs via their ability to protect the native conformation of proteins. A number of studies have revealed that organic osmolytes can interact with the peptide backbone to stabilise proteins^75–79^ and force proteins in the native conformation to fold^78,80^. Given that UVB irradiation can cause protein denaturation^81,82^, which might affect TJ proteins localisation, it is possible that the presence of organic osmolytes stabilizes the TJ proteins or counteracts any possible UVB-induced post-translational modifications of proteins. Further work is needed to explore the potential osmolyte-TJ protein interaction following UVB exposure.

Osmolytes are common constituents of skin care products due to their humectant effects. Based on the findings of this study, it is possible that organic osmolytes play other important roles, potentially including protection of developing TJs. These findings may be of great importance in furthering our understanding of the control of the epidermal barrier function, especially following exposure to UV. Our data suggest a potential role for osmolytes in sunscreens as a method to protect the development of the paracellular barrier. Since correct development and functioning of TJs maybe essential for overall barrier homeostasis^44,83,84^, the role of osmolytes may extend beyond the TJ, and osmolytes may be important to the barrier as a whole following interaction with UVR.

## Materials and Methods

### Keratinocyte culture

A continuous cell line of rat epidermal keratinocytes (REKs) (the kind gift of Professor Dale Laird, The University of Western Ontario, USA) originally isolated by Baden and Kubilus (47) was cultured as previously described in Dulbecco’s modified Eagle’s medium (DMEM, Gibco) supplemented with 10% (v/v) fetal bovine serum (FBS, HyClone), 1% (v/v) Penicillin/Streptomycin (Sigma Aldrich), 2% (v/v) L-Glutamine (Sigma Aldrich) and and 0.1 mM non-essential amino acids (Sigma Aldrich). The cells were then detached from the culture flasks with 0.25% (v/v) trypsin and 1% (v/v) ethylenediaminetetraacetic acid (EDTA, Sigma Aldrich) and were used to seed Thincert^TM^ cell culture insert with a pore size of 0.4 μm (Greiner) or on ibiTreat µ-Dish (ibidi).

### Irradiation of REKs with UVB

REKs were seeded on Thincert^TM^ cell culture inserts at approximately 10^5^cells/cm^2^ and grown until confluent, then exposed to 10 mJ/cm² UV dose in 0.5 mL phosphate buffered saline (PBS) using a single 20 W Phillips TL-12 fluorescent tube emitting 280–400 nm (peak 313 nm). After irradiation, PBS was replaced with fresh DMEM culture medium, or fresh DMEM medium supplemented with 5 mM organic osmolytes (betaine, taurine and *myo*-inositol).

### Treatment of cells with hydrogen peroxide

Confluent REKs grown on Thincert^TM^ cell culture inserts were washed with PBS and 1 mM hydrogen peroxide (H_2_O_2_) solution was prepared in phenol red-free Hank’s Balanced Salt Solution (HBSS), was added to the cells. In other experiments, the 1 mM H_2_O_2_ solution was supplemented with 5 mM betaine, taurine or myo-inositol and applied to the cells. Plates were kept at 37 °C and 5% CO2- 95% air for 1 hour, after which cells were harvested for downstream applications. The cells were incubated under the same conditions for 3 hours for immunoblotting and quantitative PCR.

### Inducing hyperosmotic stress

A hyperosmotic culture medium of 540 mOsm was prepared by adding 220 mM mannitol to DMEM culture medium (320 mOsm). Osmolarity of the culture medium was measured using an automatic cryoscopic osmometer (OSMOMAT O30, Gonotec, Berlin, Germany). The hypertonic medium was applied to confluent REK cells plated on Thincert^TM^ culture inserts and in some experiments, the hyperosmotic medium was supplemented with 5 mM of betaine, taurine or myo-inositol. For all experiments, plates were incubated for 24 hours prior to downstream applications.

### Transepithelial electrical resistance measurement

Transepithelial electrical resistance (TEER) of REKs grown on Thincert^TM^ cell culture insert was measured using an Evometer fitted with ‘chopstick’ electrodes (World Precsion Instruments; Herts, UK). TEER values (in Ω.cm^2^) were calculated by subtracting resistance values of blank Thincert™ (without cells) from the values obtained with the REK monolayers, and normalizing to the growth area. TEER was measured over 8 days in non-irradiated REK monolayers in order to confirm that the cell line could establish a paracellular barrier.

### Dextran permeability

Dextran permeability was measured on REKs grown on Thincert^TM^ cell culture insert. Cells were washed twice with HBSS and then 0.5 mL of HBSS containing FITC-Dextran (4 kD; 2 mg/ml final concentration) was added to the upper compartment and 1.5 mL of HBSS into lower compartment. Cells were incubated for 2 hours at 37 °C. FITC-Dextran concentration from each lower compartment was determined using a CLARIOstar® High Performance Monochromator microplate reader (BMG LABTECH, Ortenberg, Germany) with excitation and emission wavelengths of 485 nm and 530 nm, respectively. An apparent permeability coefficient (P_app_) was calculated following equation (1) ^85^:

1 $${P}_{app}=\,\frac{{\rm{\Delta }}Q}{{\rm{\Delta }}t}\times \frac{1}{A\times {C}_{i}\times 60}(cm/s)$$

where ∆Q/∆t is permeability rate of dextran (µg/min), A is the surface area of the filter (cm^2^), C_i_ is the initial concentration (µg/ml) and 60 is the conversion from minutes to seconds. The time point for measurement of dextran permeability was chosen based on the immunofluorescence data, which showed disruption of TJ structures 24 hours post UVB exposure.

### Immunofluorescence

REKs were grown on ibiTreat µ-Dish (ibidi) and stained at 24 hours after UV irradiation. Cells were fixed with methanol: acetone at −20 °C then permeabilised with 0.5% Triton X-100 at room temperature, then blocked using 1% (w/v) bovine serum albumin and 10% (v/v) normal goat serum solution for 1 hour. After blocking, cells were incubated with the corresponding antibody overnight at 4 °C. The primary antibodies (Life Technologies) were diluted in block solution as follows: rabbit anti-claudin- 1 (MH25, 1:50), mouse anti-claudin-4 (3E2C1, 1:50) and mouse anti-occludin (OC-3F10, 1:50). Cells were then washed with TBS and labelled with a goat anti-mouse IgG conjugated to Alexa Fluor488 (Life Technologies) for 1 hour at room temperature. The nuclei were then stained by incubation for 1 min with 4′,6-diamidino-2-phenylindole (DAPI) (Sigma Aldrich). Finally cells were mounted on petri dishes using Fluoromount G mounting medium (SouthernBiotech) and stored at 4 °C in the dark to be analysed later using a fluorescence microscope.

### RNA extraction and quantitative reverse transcription polymerase chain reaction (qRT-PCR)

Total RNA was extracted from cells using the Qiagen RNeasy Minikit (Qiagen, Manchester, UK) following the manufacturer’s instructions. cDNA was synthesized from 1 μg of total RNA using the cloned AMV first-strand cDNA synthesis kit (Invitrogen, Paisley, UK). qPCR was performed using Taqman^®^ gene expression assays (Applied Biosystems, Warrington, UK) (Pre-developed Taqman probes are provided in Table S1). Reactions were performed and analyzed using the StepOne Plus Real-Time PCR system and associated software (Applied Biosystems, Paisley, UK). Relative expression was determined against the housekeeping gene glyceraldehyde-3-phosphate dehydrogenase (GAPDH).

### Protein extraction and immunoblotting

Total protein was extracted from REK cells grown on Thincert™ cell culture inserts (Greiner Bio-one, Stonehouse, UK). The extracts were electrophoresed through precast 4–12% NuPAGE® Bis-Tris polyacrylamide gels (Novex, Life Technologies, Warrington, UK) using a 1 × NuPAGE® MES SDS running buffer (Novex, Life Technologies, Warrington, UK). Following electrophoresis, resolved proteins were electrophoretically transferred nitrocellulose membranes (Amersham, GE Healthcare, Little Chalfont, UK) using a 1 × NuPAGE® transfer buffer (Novex, Life Technologies, Warrington, UK). Membranes were then blocked for 1 hour, and then incubated with the specific primary antibody on a slow shaker overnight at 4 °C with gentle agitation. For the assessment of TJ proteins the primary antibodies (from Life Technologies, Warrington, UK): mouse anti-claudin-1 (1:1000), mouse anti-claudin-4 (1:1000), rabbit anti-occludin (1:500) were used and for the assessment of the house keeping gene the mouse anti-β-actin (1:10000) was used.

### Measurement of intracellular reactive oxygen species levels in cells

The levels of ROS were determined by cellular 2′,7′-dichlorofluorescin diacetate (DCFH-DA) as previously described^86^. Briefly, confluent REKs grown in black-walled 96-well plates were incubated with 10 μM DCFH-DA, prepared in phenol red-free HBSS, in the dark for 30 min at 37 °C. DCFH-DA was removed and fresh phenol red-free HBSS was added to the wells. DCF-DA fluorescence was measured using a CLARIOstar® High Performance Monochromator microplate reader (BMG LABTECH, Ortenberg, Germany). The excitation and emission filters were set at 485 nm and 530 nm wavelengths, respectively. The fluorescence from each well was measured, and recorded using MARS (BMG LABTECH, Ortenberg, Germany) data analysis software.

The relative percentage fluorescence increase per well was calculated using equation (2):

2 $$\frac{{(F{t}_{x}-F{t}_{0})}_{sample}}{{(F{t}_{x}-F{t}_{0})}_{control}}\times 100\,$$

where Ft_x_ is fluorescence at time x min and Ft_0_ is fluorescence at time zero min

### Cell volume measurement

Cell volume measurement was performed as previously described by Calloe *et al*.^87^. Briefly, REKs were seeded at very low density (10^3^cells/cm^2^) in ibiTreat petri dish (ibidi, Munich, Germany). REKs were irradiated as described earlier and after irradiation fresh medium or fresh culture medium supplemented with 5 mM organic osmolytes betaine, taurine or *myo-*inositol was added to cells. Plates were incubated for 24 hours, after which they were loaded with 5 µM calcein-AM (acetoxymethylester) (Molecular Probes, Oregon, United States) in PBS for 15 minutes at 37 °C. Petri dishes were washed with PBS and incubated in 300 mOsm Krebs-Henseleit (KH) buffer or KH buffer supplemented with 5 mM organic osmolytes for 30 minutes at 37 °C in order to allow de-esterification of the dye. Changes in cell volume were measured at the desired time point using a Tandem Head Leica SP5 confocal microscope equipped with a 63 × NA 1.2 objective and an argon laser. Selected cells were scanned in the x–z direction (side-view/cross-section) and images captured. The relative cell volume was calculated as the area of calcein fluorescence at time t (after the change in osmolarity), relative to the average initial area (isoosmotic, before perfusion with hyperosmotic KH buffer) using imageJ software (NIH; Bethesda, MD, USA).

### Statistical analysis

Differences were considered statistically significant at the 95% or 99% confidence level with P values calculated by the analysis of variance using One-way ANOVA test for comparisons on more than two samples, and Student’s unpaired t-test for comparisons of two samples. All statistical tests were carried out using GraphPad Prism 6 software (GraphPad Software Inc., La Jolla, CA, USA).

## Electronic supplementary material

Supplementary Information
