# Author Correction: Organic osmolytes preserve the function of the developing tight junction in ultraviolet B-irradiated rat epidermal keratinocytes

**DOI:** 10.1038/s41598-020-65508-w

**Published:** 2020-05-20

**Authors:** Cécile El-Chami, Iain S. Haslam, Martin C. Steward, Catherine A. O’Neill

**Affiliations:** 1 School of Biological Sciences, Division of Musculoskeletal & Dermatological Sciences, Faculty of Biology, Medicine and Health, University of Manchester, Oxford Road, Manchester, M13 9PT United Kingdom; 2 School of Medical Sciences, Division of Diabetes, Endocrinology and Gastroenterology, Faculty of Biology, Medicine and Health, University of Manchester, Oxford Road, Manchester, M13 9PT United Kingdom; 3 Present Address: Department of Biological Sciences, School of Applied Sciences, University of Huddersfield, Queensgate, Huddersfield HD1 3DH United Kingdom

Correction to: *Scientific Reports* 10.1038/s41598-018-22533-0, published online 26 March 2018

This Article contains errors. The panel for Claudin-4, isotonic, in Figure 4a was inadvertently duplicated from the panel for Claudin-4, control, in Figure 4d. The correct Figure 4 appears below as Figure 1.

**Figure 1:**
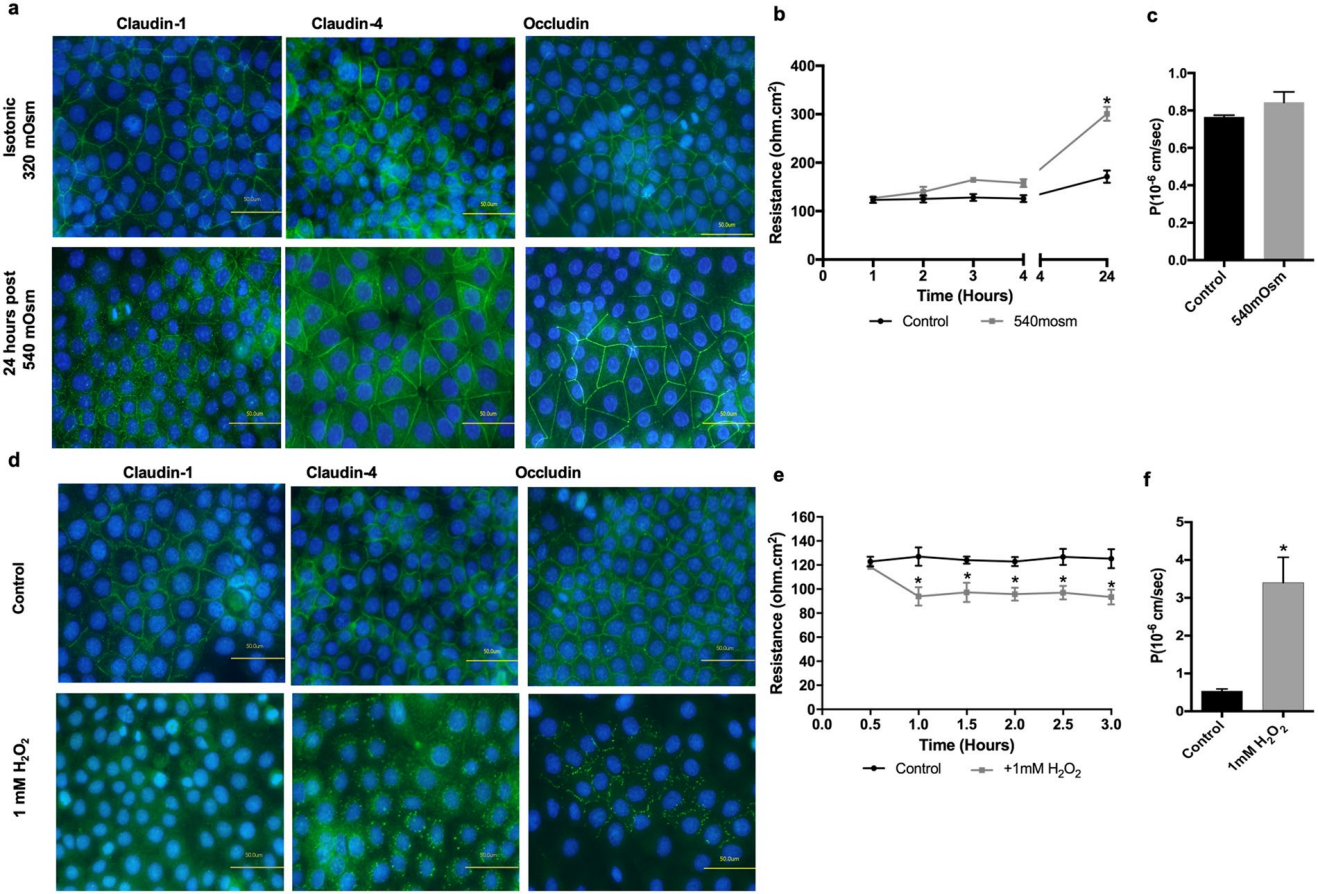
.

In addition, the TAUT panel in Supplementary Figure 2 is incorrect. The correct Supplementary Figure 2 appears below as Figure 2.

**Figure 2:**
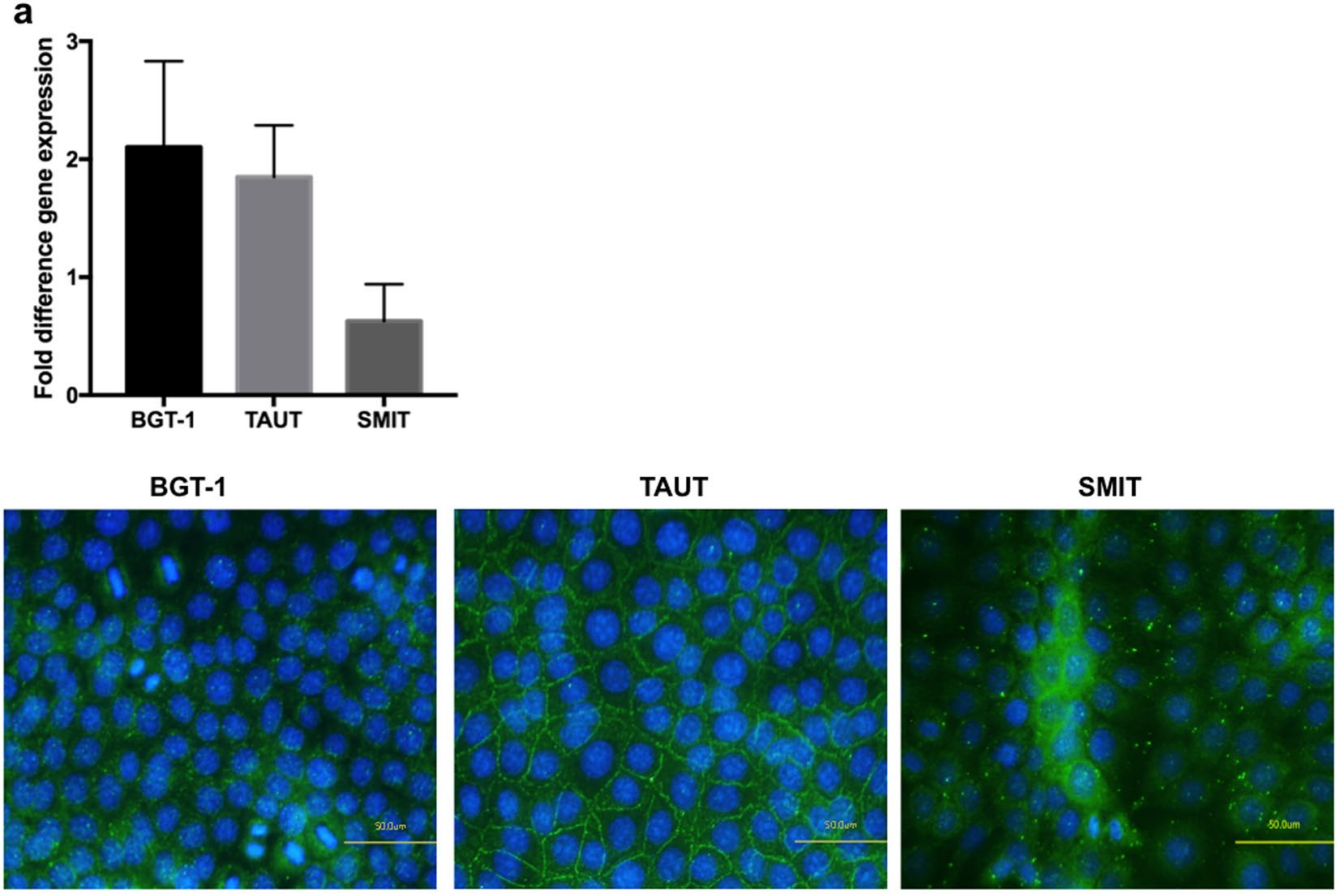
.

