# Supplementary material for: Organic osmolytes preserve the function of the developing tight junction in ultraviolet B-irradiated rat epidermal keratinocytes
Source: Sci Rep. 2018 Mar 26;8:5167. doi: 10.1038/s41598-018-22533-0 (PMC5979960; doi:10.1038/s41598-018-22533-0)
Supplement: Supplementary file 1 — Supplementary Information [file 41598_2018_22533_MOESM1_ESM.pdf]

**SUPPLEMENTARY INFORMATION**

# Organic osmolytes preserve tight junction function in ultraviolet B-irradiated rat epidermal keratinocytes

Cécile El-Chami[1], Iain S. Haslam[1,2], Martin C. Steward[3] and Catherine A. O'Neill[1]*

## MATERIALS AND METHODS:

### qRT-PCR probes

The cDNA levels were examined using pre-developed Taqman probes (Table S1).

**Table S1: List of predesigned TaqMan Gene Expression Assays used in qRT-PCR.**

| Gene Name | Species | Cat. No. | Supplier |
|---|---|---|---|
| SLC6A12 (BGT-1) | Human | Hs00758246_m1 | Thermo Fisher Scientific |
| SLC6A6 (TAUT) | Human | Hs00161778_m1 | Thermo Fisher Scientific |
| SLC5A3 (SMIT) | Human | Hs00272857_s1 | Thermo Fisher Scientific |
| GAPDH | Human | Hs02758991_g1 | Thermo Fisher Scientific |
| CLDN-1 | Rat | Rn00581740_m1 | Thermo Fisher Scientific |
| CLDN-4 | Rat | Rn01196224_s1 | Thermo Fisher Scientific |
| OCLN | Rat | Rn00580064_m1 | Thermo Fisher Scientific |
| SLC6A12 (BGT-1) | Rat | Rn00569158_m1 | Thermo Fisher Scientific |
| SLC6A6 (TAUT) | Rat | Rn00567962_m1 | Thermo Fisher Scientific |
| SLC5A3 (SMIT) | Rat | Rn01477225_s1 | Thermo Fisher Scientific |
| Cat | Rat | Rn00560930_m1 | Thermo Fisher Scientific |
| SOD1 | Rat | Rn00566938_m1 | Thermo Fisher Scientific |
| GPx1 | Rat | Rn00577994_g1 | Thermo Fisher Scientific |
| GAPDH | Rat | Rn01775763_g1 | Thermo Fisher Scientific |

### MTT assay

Cell viability after UV irradiation was assessed by 3-[4, 5-dimethylthiazol- 2-yl)]-2,5-diphenyl tetrazolium bromide (MTT). Medium containing 10% MTT stock solution (5mg/ml) in PBS was added to cells and incubated for 2 hours at 37$^{o}$C. Medium was then replaced by 500µl of dimethyl sulphoxide (DMSO) and the plates were shaken for 15 minutes. The absorbance of each well was measured at a wavelength of 540nm using a Labsystems multiscan® MC spectrophotometer (LabSystems; Finland).

**Cell volume measurement**

Cell volume measurement was performed as previously described by Calloe et al[1]. Briefly, REKs were seeded at very low density ($10^3$cells/$cm^2$) in ibiTreat petri dish (ibidi, Munich, Germany). Hypertonic culture medium was prepared by adding 220 mM Mannitol to the isotonic (320 mOsm) culture medium. Cells were incubated for 24 hours, after this time they were incubated with 5µM calcein-AM (acetoxymethylester) (Molecular probes, Oregon, United States) in PBS for 15 minutes at 37$^o$C. Petri dishes were washed with 540 mOsm Krebs-Henseleit buffer and incubated for 30 minutes at 37$^o$C in order to allow de-esterification of the dye.

## RESULTS

### Effect of UVB on cell viability

In the first experimental set-up, irradiation doses ranging from 0 to 40 mJ/cm$^2$ were used to identify sub-lethal doses of UVB. Twenty-four hours post-irradiation, the viability of the cells was measured using an MTT assay (Figure S1). Because 20, 30 and 40 mJ/cm$^2$ caused more than 50% dead cells compared to 13% caused by 10mJ/cm$^2$, this latter dose was chosen for further experiments.

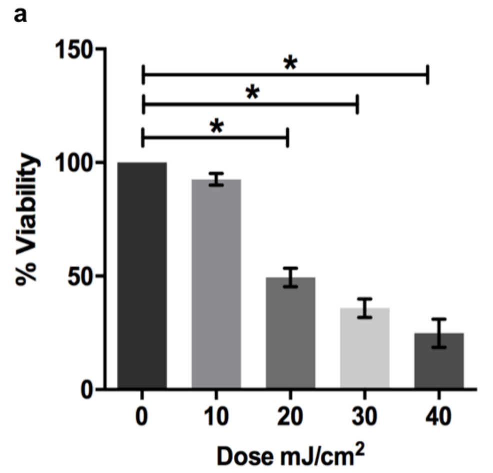


**Figure S1: UVR doses higher than 10mJ/cm$^2$ significantly affect REK viability**
Monolayers of REKS were irradiated with various doses of UVB ranging from 0-40mJ/cm$^2$ and cell viability was measured 24 hours post-irradiation using MTT assay. At doses of 20mJ/cm$^2$ and higher, the cell viability was significantly lower than unirradiated cells (0mJ/cm$^2$) (*: P<0.001; n=6).

**Expression of organic osmolytes transporters in REKs**

The expression profile of BGT-1, TAUT and SMIT in REKs was compared with that of the human primary keratinocytes, which according to literature are known to express these organic osmolytes transporters[2,3]. Quantitative PCR analysis of RNA extracted from REKs showed that BGT-1, TAUT and SMIT are expressed at the gene level (Figure S2a). In addition, the subcellular localisation of the organic osmolyte transporters BGT-1, TAUT and SMIT in REKs was in line with what has been found in other cell lines. Similar to MDCK cells in isotonic conditions[4], REKs were found to express BGT-1 in the cytoplasm (Figure S2b). The taurine transporter TAUT was found to be localised both in the cytoplasm and cell membrane of mouse fibroblasts[5] and mouse neural precursor cells[6]. In accordance with these previous findings, our data showed that in REKs TAUT was expressed at the plasma membrane (Figure S2b). Similar to BGT-1, SMIT was found to be localised in cytosolic vesicles in human embryonic kidney cells[7]. Our data also showed that SMIT in REKs was localised in the cytoplasm (Figure S2b).

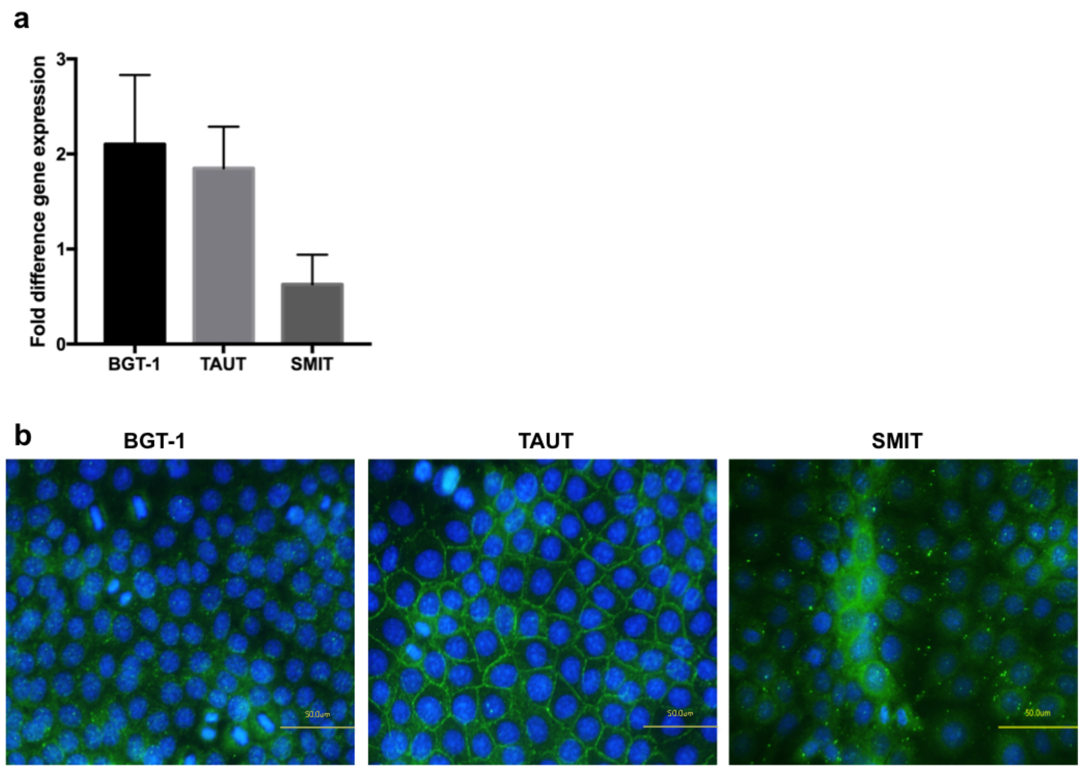


**Figure S2: Organic osmolytes transporters expression in REKs**
(a) Relative organic osmolytes transporter gene expression levels in REKs, relative to NHEKs. The normalised expression levels in REKs are expressed relative to total RNA from NEHKs. Positive fold values indicate organic osmolytes genes expressed at a higher level than NHEKs (n = 4; mean ± SEM). (b) Immunostaining of BGT-1, TAUT and SMIT in normal cultures of REKs. Bar = 50μm.

**Effect of organic osmolytes on control, non-irradiated REKs**

Treatment of non-irradiated REKs with 5 mM organic osmolytes resulted in no changes in TEER (Figure S3a) and dextran permeability (Supplementary Fig. S3b).

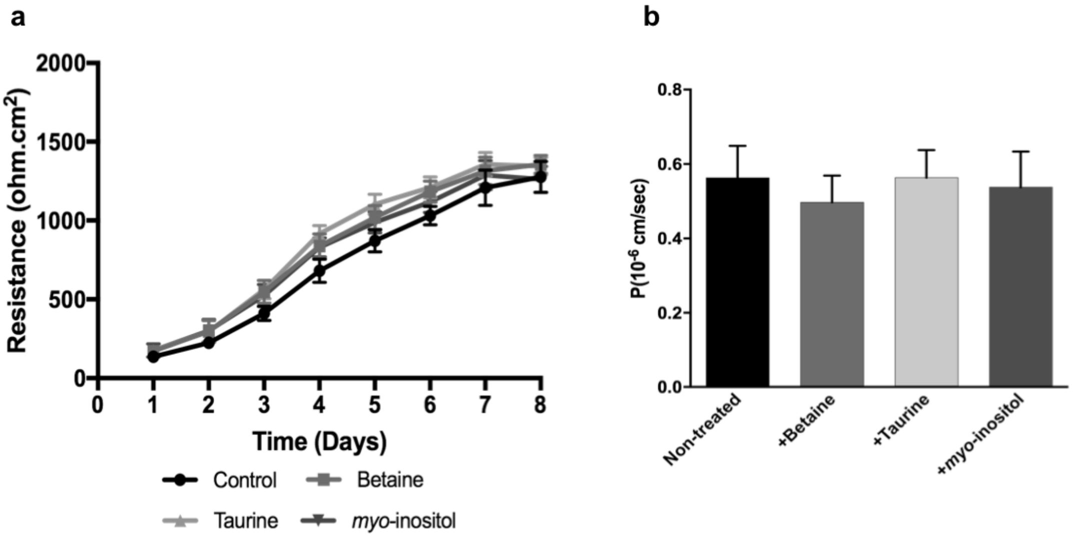


**Figure S3: Effect of organic osmolytes on tight junction function in REKs.**
(**a**) TEER measurements following organic osmolytes treatment (n = 5; mean ± SEM). (**b)** Permeability of 4 kDa FITC-dextran (FD4) 24 hours after treatment with 5 mM organic osmolytes (n = 5; mean ± SEM).

### Effect of UVB on TJ proteins gene expression

REKs were exposed to a single dose of UVB and qRT-PCR analysis of claudin-1, claudin-4, and occludin was investigated 24 hours later (Figure S4). The data indicated no change in the mRNA of all tested TJ proteins in irradiated REKs compared to non-irradiated cells. Moreover, supplementation with organic osmolytes (5 mM betaine, taurine or *myo*-inositol) had no effect on the mRNA levels of the three TJ proteins in irradiated REKs.

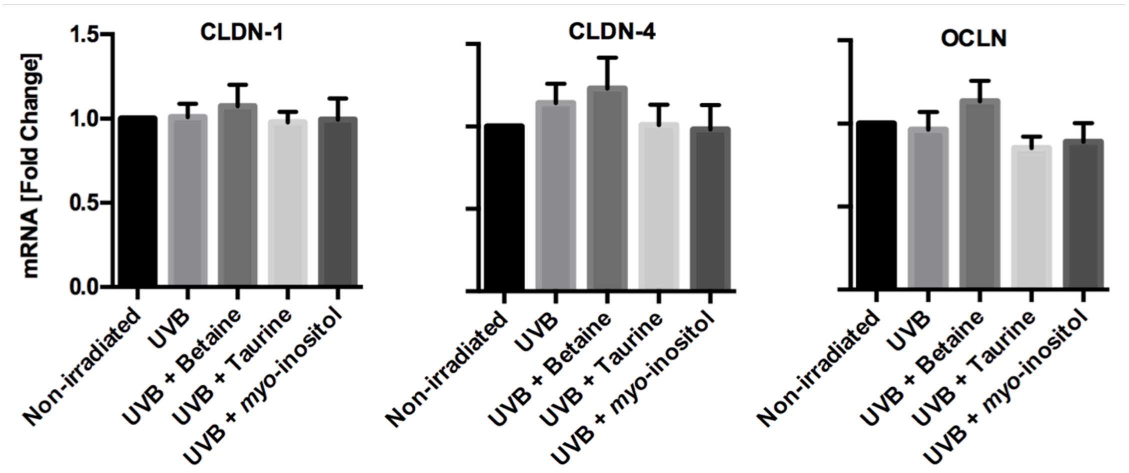


**Figure S4: The effect of organic osmolytes on tight junction protein gene expression after UVB irradiation.**
Organic osmolytes were supplemented to the culture medium directly after UVB irradiation. qRT-PCR analyses showed no change in the gene expression levels of claudin-1, -4, and occludin in UVB-irradiated REKs in the presence and absence of 5 mM organic osmolytes (n = 3; mean ± SEM).

# Supplementary Figure S5. Full-size blots of Fig. 3b

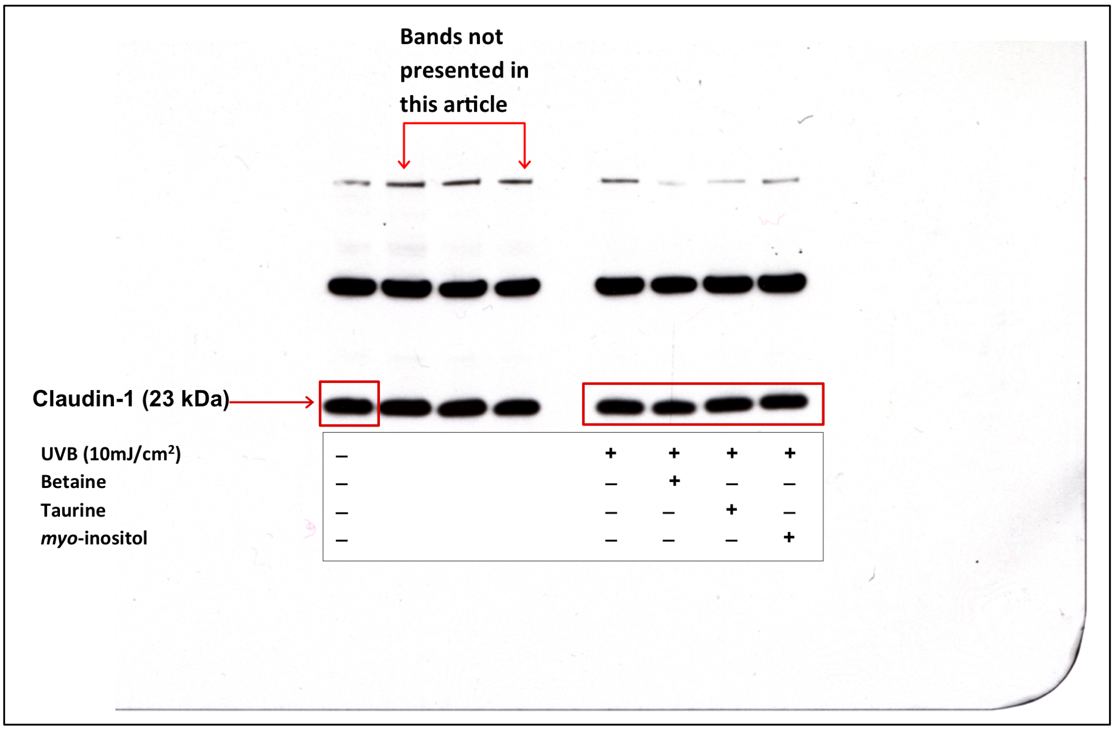


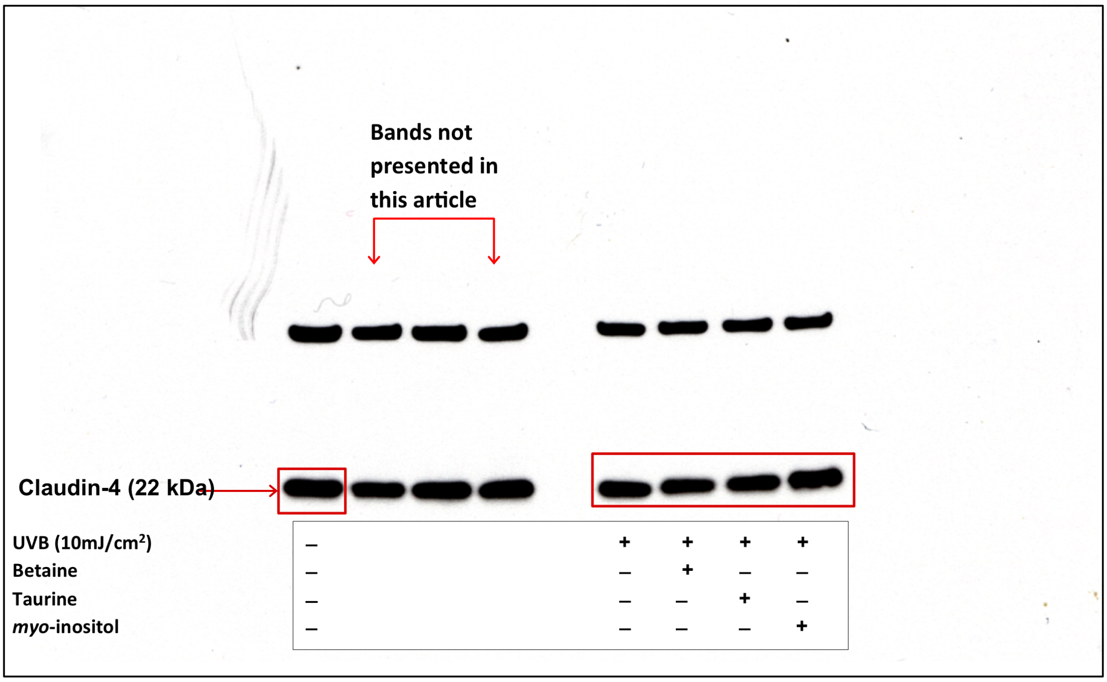

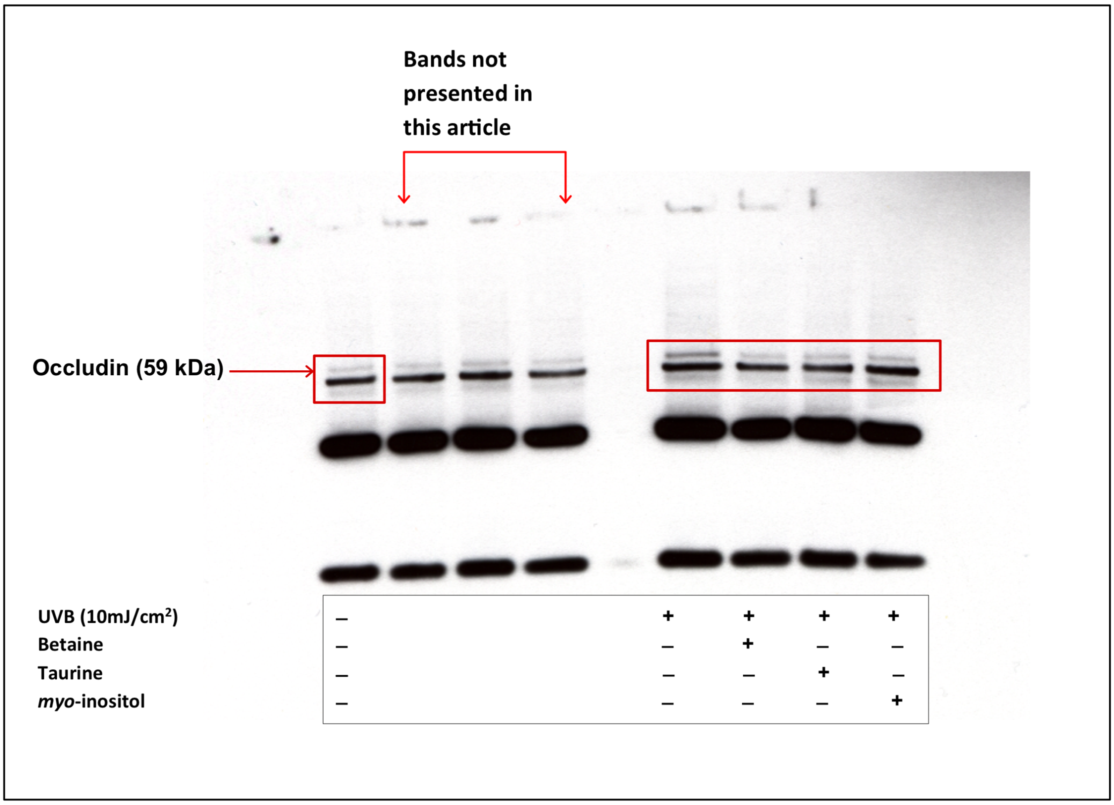
Bands not presented in this article
Occludin (59 kDa)
UVB (10mJ/cm²)
Betaine
Taurine
myo-inositol
– + + + +
– – + – –
– – – + –
– – – – +

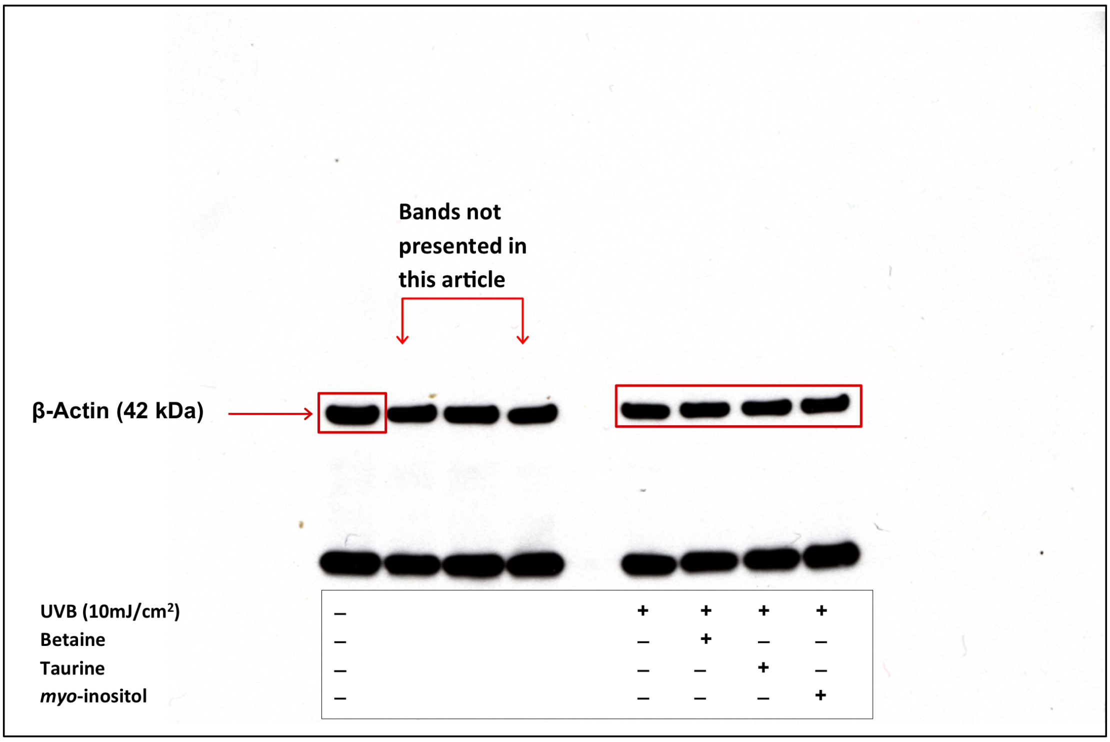
Bands not presented in this article
β-Actin (42 kDa)
UVB (10mJ/cm²)
Betaine
Taurine
myo-inositol
– + + + +
– – + – –
– – – + –
– – – – +

**Effect of hyperosmotic stress on tight junction gene and protein expression**

REKs shrink after exposure to 540 mOsm culture medium (Figure S6a & b). Claudin-1 and occludin gene (Figure S6c) and protein expression (Figure S6d &e) were not affected by hypertonic stress, while claudin-4 showed an upregulation at the gene (Figure S6c) and protein levels (Figure S6d & e).

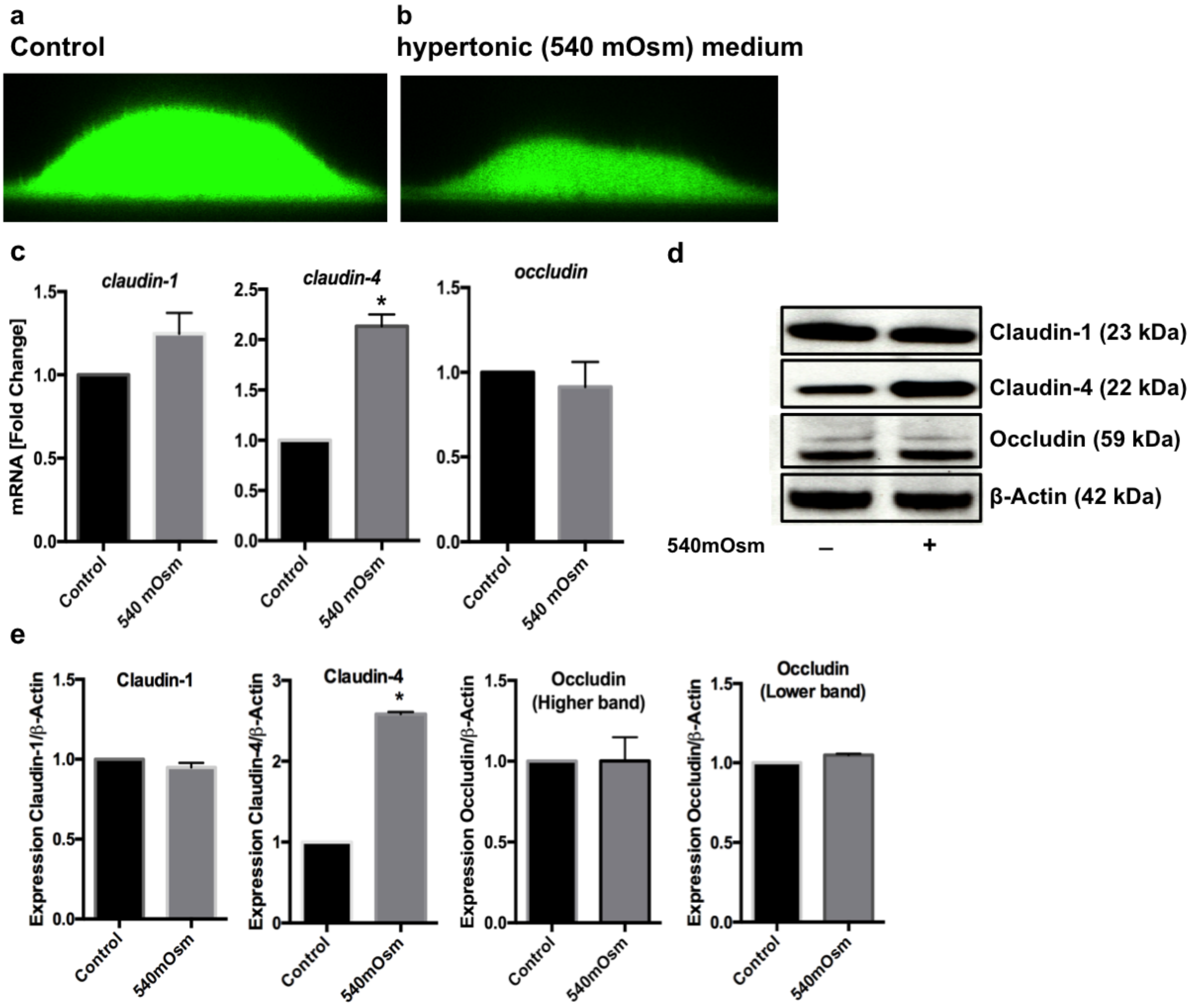


**Figure S6: Effect of hyperosmotic stress on tight junction gene and protein expression.**
(a) Images represent REKs under control isotonic culture conditions and (b) post exposure to hyperosmotic culture medium. (c) qRT-PCR analyses (n = 3; mean ± SEM; *: $p<0.05$ compared to control condition) and (d) western blot analyses were performed after 24 hours of hypertonic stress in the presence and absence of organic osmolytes (5 mM). Full-length blots are presented in supplementary Fig. S6. (e) Densitometric quantification showing the ratio of TJ proteins to ß-actin protein expression (n = 3; mean ± SEM; *: $p<0.05$ compared to control condition).

**Supplementary Figure S7. Full-size blots of Supplementary Fig. S6**

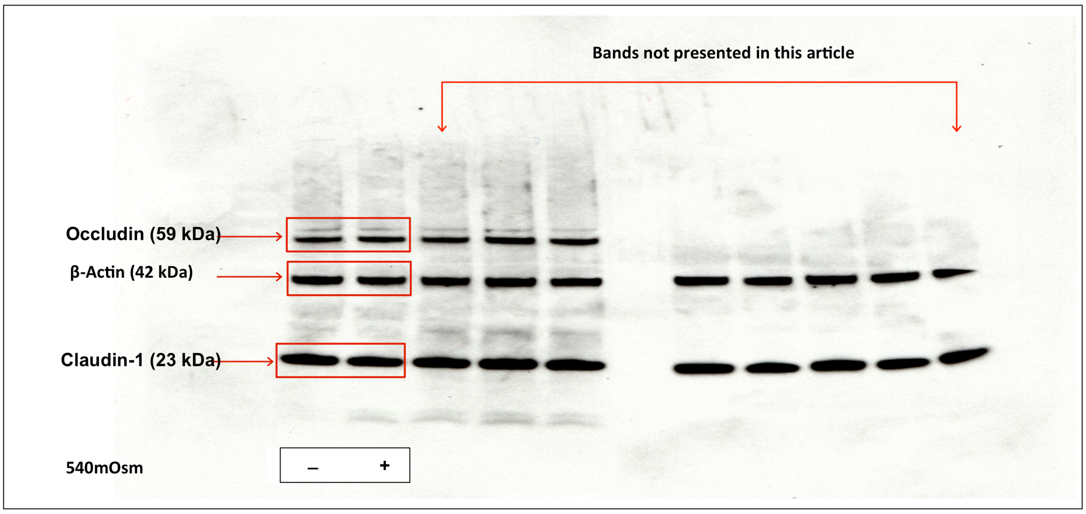


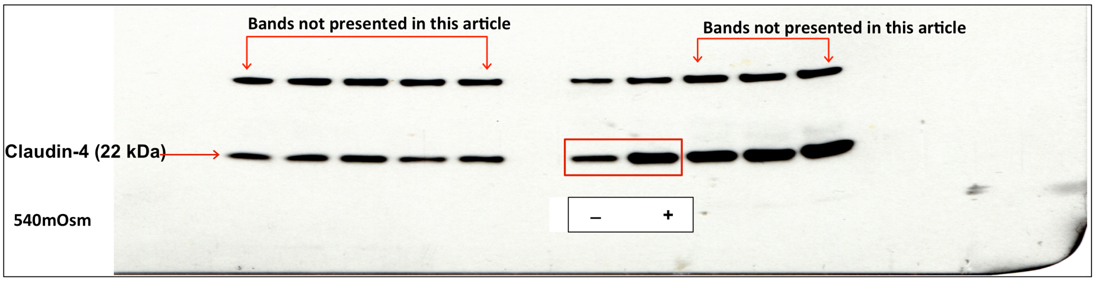


### $H_2O_2$ effect on the gene and protein levels in REKs

REKs were exposed to $H_2O_2$ and qRT-PCR and western blot analyses were performed after 3 hours. Claudin-1, claudin-4 and occludin mRNA expression decreased after $H_2O_2$ exposure and the addition of organic osmolytes had no change at the gene level (Figure S8a). Claudin-1 protein expression decreased after $H_2O_2$ application and it was not affected by treatment with organic osmolytes (Figure S8 b&c). Occludin showed an increase in the higher molecular weight band intensity following treatment with taurine (Figure S8 b&c).

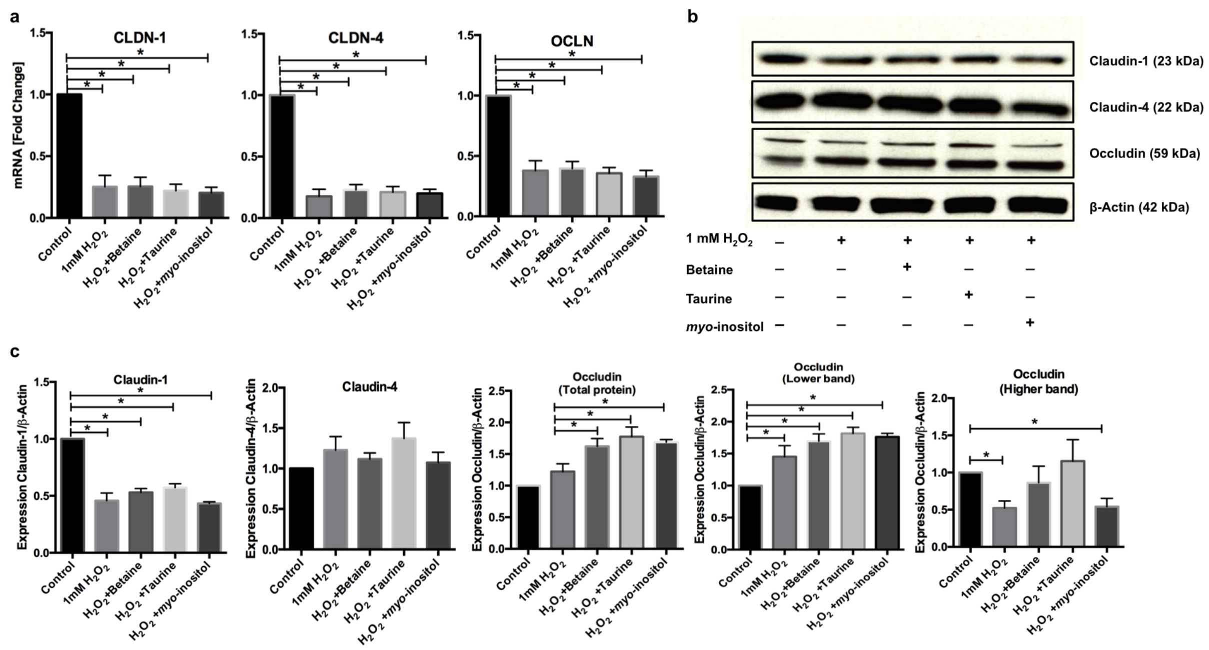


**Figure S8: Effect of $H_2O_2$ on TJ gene and protein expression**

(a) qRT-PCR analysis of claudin-1, claudin-4 and occludin mRNA expression following $H_2O_2$ exposure with and without the addition of organic osmolytes. (b) Western blot analysis of TJ protein expression following $H_2O_2$ exposure with and without organic osmolytes. Full-length blots are presented in supplementary Fig. S8. (c) Densitometric quantification showing the ratios of TJ proteins to ß-actin protein expression (n = 3; mean ± SEM; *: $p<0.05$ compared to sample exposed to $H_2O_2$ and non-supplemented with organic osmolytes).

**Supplementary Figure S9. Full-size blots of Supplementary Fig. S8**

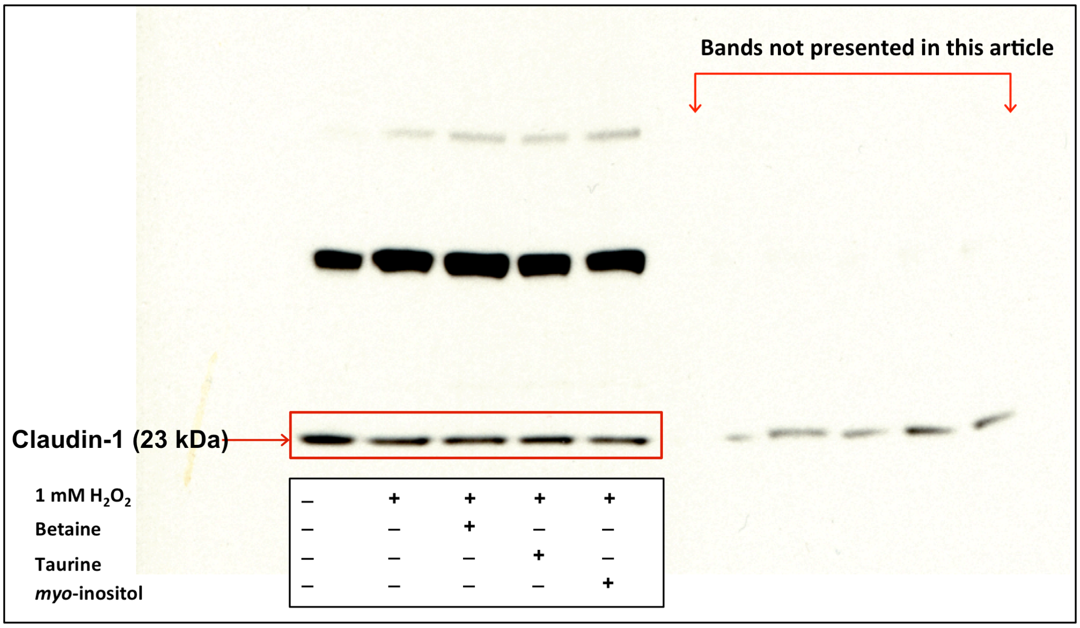


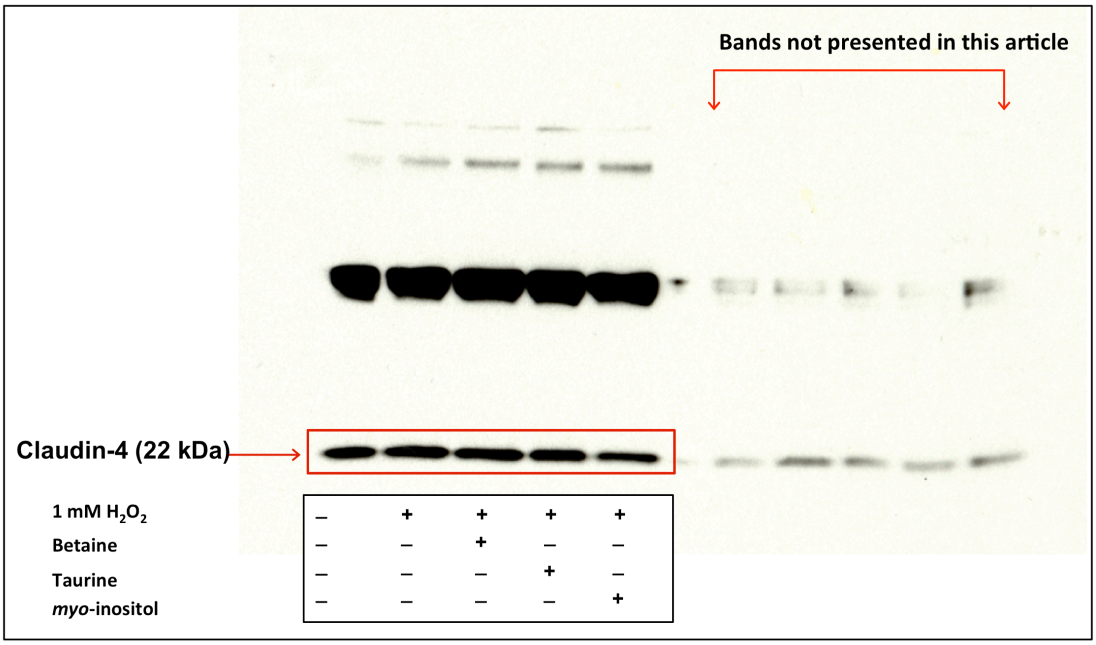

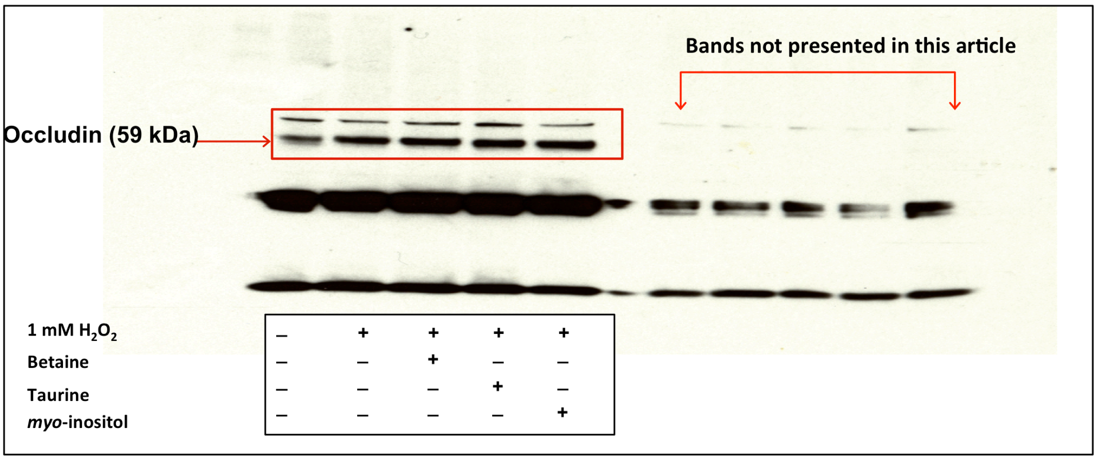


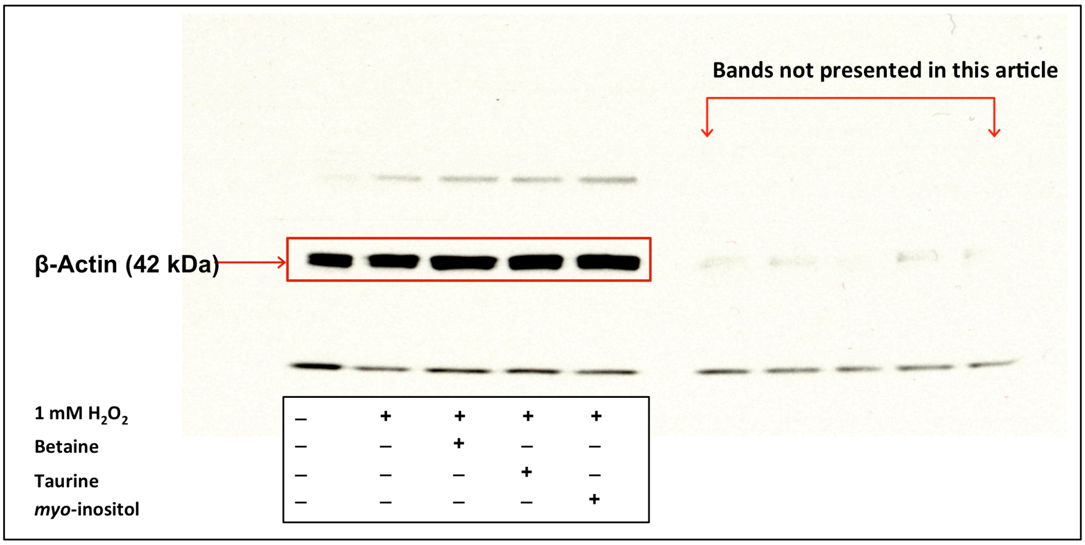


**ROS measurement in REKs following exposure to $H_2O_2$**

Cells were loaded with DCFH-DA and fluorescence intensity measurement initiated immediately after the application of $H_2O_2$ by placing the plate directly in the plate reader and fluorescence measurements were taken in real time for 1 hour.

Cells showed a 374.81 ± 68.07% increase in DCF fluorescence intensity 1 hour post exposure to $H_2O_2$ (Figure S10a). Interestingly, in cells cultured with 5 mM betaine, taurine or *myo*-inositol a similar increase in fluorescence intensity was recorded.

The mRNA levels of the antioxidant enzymes catalase (Cat), superoxide dismutase (SOD1) and glutathione peroxidase (GPx1) were investigated in REKs 3

hours after exposure to 1 mM $H_2O_2$ (Figure S10b). Cat mRNA levels were not significantly different in $H_2O_2$ exposed cells compared to control cells. In contrast a significant increase in SOD1 and GPx1 mRNA levels was observed 3 hours after exposure to 1 mM $H_2O_2$. The presence of organic osmolytes had no effect on the elevation in gene expression of SOD1 and GPx1 caused by ROS production.

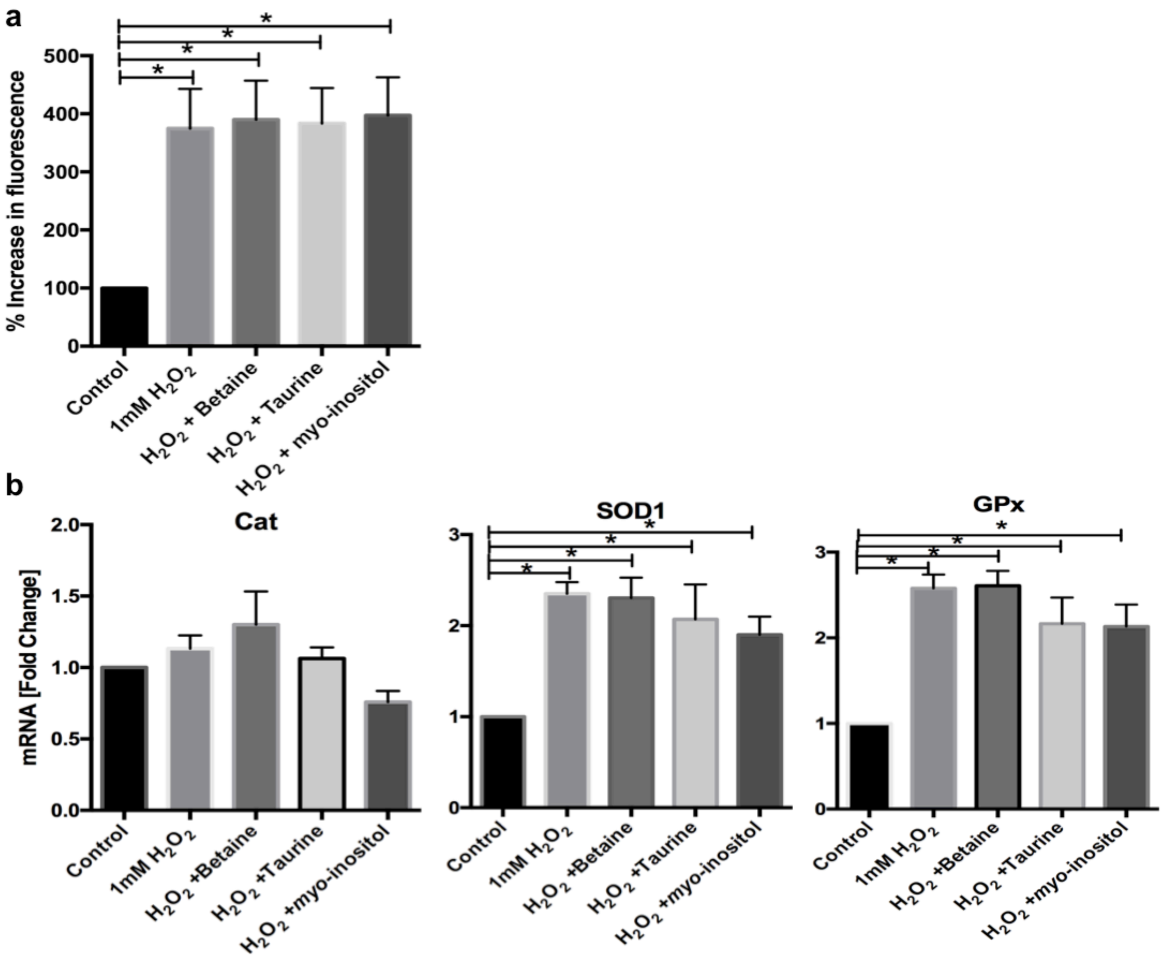


**Figure S10: ROS production and antioxidant gene expression after $H_2O_2$ exposure.**
(a) An increase in DCF fluorescence corresponded to an increase in intracellular ROS (n = 3; mean ± SEM; *: $p<0.05$ compared to cells exposed to $H_2O_2$ and non-supplemented with organic osmolytes). (b) Cat, SOD1 and GPx1 gene expression levels were significantly higher, in $H_2O_2$-exposed with and without organic osmolyte supplementation (n = 3; mean ± SEM; *: $p<0.05$ compared to cells exposed to $H_2O_2$ and non-supplemented with organic osmolytes).

**REFERENCES:**


1. Calloe, K., Nielsen, M. S., Grunnet, M., Schmitt, N. & Jorgensen, N. K. KCNQ channels are involved in the regulatory volume decrease response in primary neonatal rat cardiomyocytes. *Biochim. Biophys. Acta - Mol. Cell Res.* **1773,** 764–773 (2007).
2. Warskulat, U., Reinen, A., Grether-Beck, S., Krutmann, J. & Haussinger, D. The osmolyte strategy of normal human keratinocytes in maintaining cell homeostasis. *J. Invest. Dermatol.* **123,** 516–521 (2004).
3. Janeke, G. *et al.* Role of taurine accumulation in keratinocyte hydration. *J. Invest. Dermatol.* **121,** 354–361 (2003).
4. Kempson, S. A., Parikh, V., Xi, L. X., Chu, S. Y. & Montrose, M. H. Subcellular redistribution of the renal betaine transporter during hypertonic stress. *Am. J. Physiol. Physiol.* **285,** C1091–C1100 (2003).
5. Voss, J. W., Pedersen, S. F., Christensen, S. T. & Lambert, I. H. Regulation of the expression and subcellular localization of the taurine transporter TauT in mouse NIH3T3 fibroblasts. *Eur. J. Biochem.* **271,** 4646–4658 (2004).
6. Hernandez-Benitez, R., Pasantes-Morales, H., Pinzon-Estrada, E. & Ramos-Mandujano, G. Functional Expression and Subcellular Localization of the Taurine Transporter TauT in Murine Neural Precursors. *Dev. Neurosci.* **32,** 321–328 (2010).
7. Andronic, J. *et al.* Hypotonic Activation of the Myo-Inositol Transporter SLC5A3 in HEK293 Cells Probed by Cell Volumetry, Confocal and Super-Resolution Microscopy. *PLoS One* **10,** (2015).
